# Good Steel Used in the Blade: Well‐Tailored Type‐I Photosensitizers with Aggregation‐Induced Emission Characteristics for Precise Nuclear Targeting Photodynamic Therapy

**DOI:** 10.1002/advs.202100524

**Published:** 2021-05-21

**Authors:** Miaomiao Kang, Zhijun Zhang, Wenhan Xu, Haifei Wen, Wei Zhu, Qian Wu, Hongzhuo Wu, Junyi Gong, Zhijia Wang, Dong Wang, Ben Zhong Tang

**Affiliations:** ^1^ Center for AIE Research Shenzhen Key Laboratory of Polymer Science and Technology Guangdong Research Center for Interfacial Engineering of Functional Materials College of Materials Science and Engineering Shenzhen University Shenzhen 518060 China; ^2^ Hong Kong Branch of Chinese National Engineering Research Center for Tissue Restoration and Reconstruction Department of Chemistry The Hong Kong University of Science and Technology Clear Water Bay Kowloon Hong Kong 999077 China; ^3^ State Key Laboratory of Fine Chemicals School of Chemical Engineering Dalian University of Technology Dalian 116024 China

**Keywords:** aggregation‐induced emission, nuclear targeting, photodynamic therapy, type‐I photosensitizer

## Abstract

Photodynamic therapy (PDT) has long been recognized to be a promising approach for cancer treatment. However, the high oxygen dependency of conventional PDT dramatically impairs its overall therapeutic efficacy, especially in hypoxic solid tumors. Exploration of distinctive PDT strategy involving both high‐performance less‐oxygen‐dependent photosensitizers (PSs) and prominent drug delivery system is an appealing yet significantly challenging task. Herein, a precise nuclear targeting PDT protocol based on type‐I PSs with aggregation‐induced emission (AIE) characteristics is fabricated for the first time. Of the two synthesized AIE PSs, TTFMN is demonstrated to exhibit superior AIE property and stronger type‐I reactive oxygen species (ROS) generation efficiency owing to the introduction of tetraphenylethylene and smaller singlet–triplet energy gap, respectively. With the aid of a lysosomal acid‐activated TAT‐peptide‐modified amphiphilic polymer poly(lactic acid)12k–poly(ethylene glycol)5k–succinic anhydride‐modified TAT, the corresponding TTFMN‐loaded nanoparticles accompanied with acid‐triggered nuclear targeting peculiarity can quickly accumulate in the tumor site, effectively generate type‐I ROS in the nuclear region and significantly suppress the tumor growth under white light irradiation with minimized systematic toxicity. This delicate “Good Steel Used in the Blade” tactic significantly maximizes the PDT efficacy and offers a conceptual while practical paradigm for optimized cancer treatment in further translational medicine.

## Graphical abstract

A precise nuclear targeting photodynamic therapy (PDT) protocol based on tactfully designed type‐I photosensitizers with aggregation‐induced emission characteristics is fabricated for the first time with the aid of a lysosomal acid‐activated TAT‐modified nuclear delivery system, which can significantly maximize the PDT efficacy.

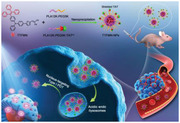

## 1 Introduction

Cancer is undeniably one of the most intricate and refractory disease with an increasing morbidity and extremely high mortality, leaving a serious threat to human health around the world.^[^ ^1^ ^]^ Nowadays, as an emerging minimally noninvasive therapeutic modality for cancer treatment, photodynamic therapy (PDT) has attracted intense research interests by virtue of its high spatiotemporal precision and accurate controllability.^[^ ^2^, ^3^, ^4^, ^5^, ^6^ ^]^ Upon light activation, excited photosensitizers (PSs) undergo type‐I (electron transfer) and/or type‐II (energy transfer) reactions^[^ ^7^ ^]^ to generate destructive reactive oxygen species (ROS) to damage exposed tumor site through inducing cell apoptosis or necrosis, vascular damage, and probably the immunity response.^[^ ^8^, ^9^ ^]^ The vast majority of PSs currently used are type‐II ones that intrinsically rely on O_2_ to produce singlet oxygen (^1^O_2_, type‐II ROS).^[^ ^10^ ^]^ Type‐II PDT has being suffered from the extreme O_2_ shortage in the ubiquitously aberrant microenvironment of solid tumors caused by the aggressive neoplastic cell proliferation and the insufficient blood supply.^[^ ^11^, ^12^ ^]^ To combat this obstacle, lots of innovative approaches aiming at increasing the intratumoral oxygen perfusion or generation have recently been proposed,^[^ ^13^, ^14^ ^]^ which are capable of partially solving such problem but often cause untoward side effects, such as hyperoxic seizures and barotrauma problems.^[^ ^15^ ^]^ Worse still, it has been demonstrated that O_2_ enriching even also promotes cancerous cell proliferation and inhibits cell apoptosis, which as a result compromises the therapeutic efficacy.^[^ ^16^ ^]^

Given the circumstances, PDT on the basis of less O_2_‐dependent type‐I PSs would be a more promising strategy for cancer treatment. Excited type‐I PSs can transfer electrons to the surrounding substrates and O_2_, yielding ROS species such as superoxide (^•^O_2_^−^) and hydroxyl (^•^OH) radicals.^[^ ^7^, ^17^ ^] •^O_2_^−^ is supposed to be the primary precursor of type‐I process that can be further transformed into more chemically reactive and highly toxic ^•^OH via secondary reactions ultimately, accompanying with the production of O_2_.^[^ ^7^, ^18^, ^19^ ^] •^OH is considered to be the most aggressive reactive oxygen centered radical in biology which can react directly with various vital biomacromolecules, consequently exerting amplified PDT response.^[^ ^20^ ^]^ Notably, the hypoxic condition in solid tumors can be partially alleviated because O_2_ is recyclable in these cascade bioreactions.^[^ ^21^ ^]^ Thus, type‐I PDT with highly reactive radicals generation and stronger hypoxia tolerance would be an appealing option to overcome the limitations of traditional type‐II PDT, which can open up a new avenue for solid tumor treatment.^[^ ^22^, ^23^ ^]^ However, suffering from the worrisome biosecurity, poor reproducibility, and complicated pharmacokinetics induced by the complex components, the reported approaches undergoing type‐I process to date, such as antennae–fullerene conjugates,^[^ ^24^ ^]^ inorganic nanocomposites,^[^ ^25^ ^]^ organometallic complexes,^[^ ^26^ ^]^ and metal–organic frameworks,^[^ ^27^ ^]^ are far from ideal for clinical application.

Recently, a few novel type‐I PSs based on purely organic small molecule bearing obvious merits of optimal biosafety, structural diversity, flexible preparation, and outstanding reproducibility have encouragingly emerged,^[^ ^28^, ^29^ ^]^ some of which, attractively, can aid in imaging‐guided PDT, since they are capable of emitting fluorescence intrinsically.^[^ ^21^, ^22^, ^23^ ^]^ However, the PDT efficiency based on these organic PSs is usually limited because most of these PSs possess hydrophobic and planar structure as well as large conjugation, and are easy to form aggregates in physiological environments of aqueous media, consequently resulting in a much weakened fluorescence emission and extremely decreased ROS generation in aggregate state caused by the strong *π*–*π* stacking and other nonradiative decay pathways.^[^ ^30^ ^]^ The exploration of a newly emerging type of PSs with aggregation‐induced emission (AIE) characteristics would be an ingenious solution. Sharply different from conventional PSs, AIE‐active PSs become highly emissive and stronger ROS‐productive in aggregate state because of their unique rotor‐like twisted structures, which lead to significantly extended intermolecular distance and reduced intermolecular *π*–*π* interaction as well as facilitated intersystem crossing (ISC) process upon aggregation.^[^ ^31^, ^32^, ^33^, ^34^, ^35^ ^]^ Notwithstanding the great potential, development of AIE PSs based on type‐I mechanism for the desired boosted PDT performance is still in its infancy and further research and immense efforts are in high demand.^[^ ^36^, ^37^ ^]^

Additionally, subcellular organelle‐targeting site of PSs is of vital importance for implementing PDT.^[^ ^38^ ^]^ Of particular interest is cell nucleus, which plays a predominant role in resisting cell death, activating invasion, and metastasis of cancer cells.^[^ ^39^ ^]^ Moreover, it has been demonstrated that DNA or RNA damage is the most direct and serious lesion type for the cytotoxicity induced by type‐I ROS relying on its highly oxidizing activity.^[^ ^40^, ^41^ ^]^ Evidently, it would be of great significance to exploit appropriate delivery systems, particularly for tumor microenvironment or intracellular stimulus‐responsive nanocarriers, to precisely transport efficient AIE‐active type‐I PSs into cancer cell nuclei for high‐performance PDT.

In this contribution, for the first time, a nucleus‐targeting PDT strategy based on type‐I AIE PSs was rationally designed and successfully developed. Two near‐infrared (NIR)‐emissive AIE luminogens (AIEgens), termed as TFMN and TTFMN, were facilely synthesized. Through refined tuning of molecular structure, TTFMN exhibited better AIE tendency, more bathochromic absorption and emission wavelengths, as well as more efficient type‐I ROS production. To maximize the PDT effect of TTFMN, a pH‐activated TAT‐peptide‐modified amphiphilic polymer was utilized as an encapsulation matrix to transport PSs precisely into cell nuclei of tumor. Both in vitro and in vivo results validated that the afforded TTFMN nanoparticles (NPs) performed well in practical biomedical application in terms of NIR‐fluorescence‐imaging‐guided, nucleus‐targeted, and type‐I‐ROS‐induced photodynamic cancer theranostics.

## 2 Results and Discussion

### 2.1 Molecular Design, Synthesis, and Characterization

Efficient ISC process is widely recognized as one of the key factors for effective ROS generation.^[^ ^42^ ^]^ The construction of molecules with high electron donor (D)–acceptor (A) strength usually represents an effective strategy to accelerate the ISC process owing to the decreased singlet–triplet energy gap (Δ*E*_s–t_) caused by separating the highest occupied molecular orbital (HOMO) and the lowest unoccupied molecular orbital (LUMO) distribution. Intriguingly, high electron affinity of PSs plays predominant role in type‐I ROS generation, in which the triplet PSs are first prone to transform into intermediate radical anions (**^•^** PS**^−^**) through accepting electrons and subsequently transfer electrons to O_2_ or other adjacent substrates, yielding type‐I ROS.

**Figure 1:**
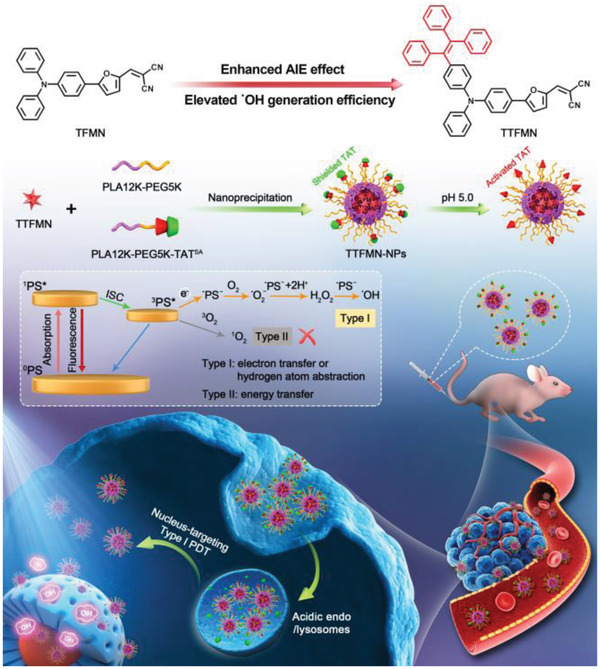
Illustration of molecular design principle of TFMN and TTFMN, photophysical and photochemical mechanisms of type‐I and type‐II processes, construction of acid‐activated TTFMN NPs, and their applications in precise photodynamic nuclear targeting cancer therapy.

As shown in **Figure** **1**, the designed compound TFMN possesses typical D–A structure with a triphenylamine (TPA) fragment serving as electron donor, a furan unit working as electron donor and *π*‐bridge, a carbon–carbon double bond working as *π*‐bridge, and two cyano units acting as electron acceptor, exhibiting strong D–A strength, which is envisioned to potentiate the ROS generation capacity of TFMN. Additionally, the twisted conformation of TPA fragment will result in the expected activation of radiative channels in the aggregate state by validly suppressing intermolecular *π*–*π* stacking, thus guaranteeing the AIE signature of TFMN. Notably, the extreme electron deficiency of two cyano units is anticipated to favor the production of type‐I ROS. Furthermore, as a star AIE‐active building block, tetraphenylethylene (TPE) is introduced into the TFMN skeleton to establish a homologous compound TTFMN. Sufficient molecular rotors of TPE could consume the exciton energy upon photoexcitation, resulting in nonemission or extremely weak emission of TTFMN in solution. Besides, coupled with the more twisted conformation of TPE, the fluorescence quenching of TTFMN aggregates will be further prevented by virtue of the further enlarged intermolecular distance and ulteriorly reduced intermolecular *π*–*π* interaction, thus potentially boosting AIE characteristics.

To verify these hypotheses, compounds TFMN and TTFMN were simply synthesized through a few reaction steps. As illustrated in Figure S1 (Supporting Information), 4‐bromo‐*N*,*N*‐diphenylaniline or presynthesized 4‐bromo‐*N*‐phenyl‐*N*‐(4‐(1,2,2‐triphenylvinyl)phenyl)aniline was primarily coupled with (5‐formylfuran‐2‐yl)boronic acid smoothly by Suzuki–Miyaura coupling reaction in the presence of Pd catalyst, giving intermediate products **1** and **4** in excellent yields. Then, Knoevenagel condensation reaction between intermediate products **1** or **4** and malononitrile was conducted to produce the designed compounds TFMN and TTFMN with the yields of 88.0% and 85.9%, respectively (Figures S2–S7, Supporting Information).

### 2.2 Photophysical Properties and Theoretical Calculations

**Figure 2:**
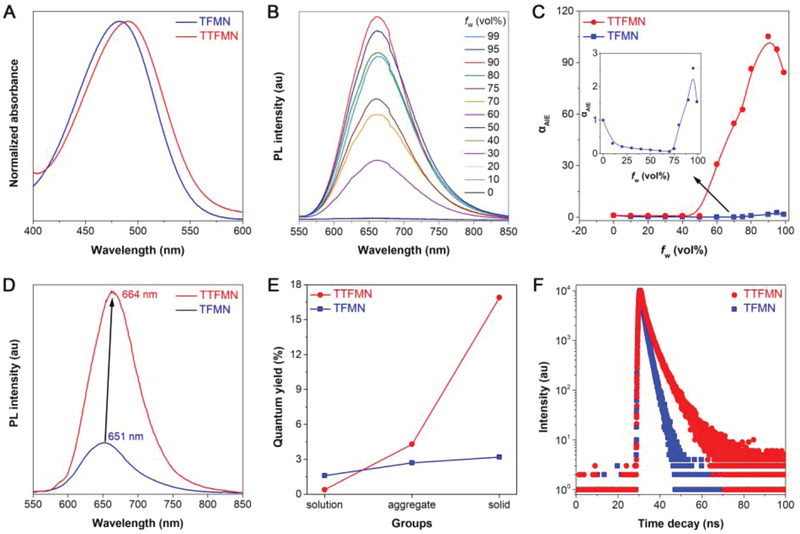
Optical properties of TFMN and TTFMN. A) Normalized absorption spectra of TFMN and TTFMN in ACN solution. B) PL spectra of TTFMN (10 × 10^−6^ m) in ACN/water mixtures with different water fractions (*f*_w_). C) The plots of the relative emission intensity (*I*/*I*_0_) of TFMN and TTFMN versus water fraction, *I*_0_ and *I* are the peak PL intensities of AIEgens (10 × 10^−6^ m) in pure ACN and ACN/water mixtures, respectively. Inset: enlarged *I*/*I*_0_ plots of TFMN. D) Normalized PL spectra of TFMN and TTFMN in the solid state. E) Quantum yields of TFMN and TTFMN measured in ACN, ACN/water mixtures (*f*_w_ = 90%), and solid state. F) Time‐resolved decay profiles of TFMN and TTFMN.

The optical properties of TFMN and TTFMN were preliminarily studied by UV–vis and photoluminescence (PL) spectroscopy, as summarized in Table S1 (Supporting Information). The absorption spectra of TFMN and TTFMN were centered at 482 and 490 nm in acetonitrile (ACN), respectively (**Figure** **2A**). Density functional theory calculation results demonstrated that the HOMO–LUMO energy gaps of TFMN and TTFMN were calculated to be 2.63 and 2.48 eV, respectively (Figure S8, Supporting Information), which were in good accordance with the redshifting absorption maxima from TFMN to TTFMN. The AIE features of TFMN and TTFMN were further evaluated by employing a mixed solvent (ACN/water) system with water used as a poor solvent. As shown in Figure 2B,C, TTFMN emitted faint fluorescence in ACN solutions (*Ф*_F, soln._ = 0.4%) and remained very weakly emissive until the water fraction increased to 50%. Further raising the water fraction to 90%, the fluorescence intensity of TTFMN increased for 105.2‐fold comparing with that of pure ACN solution, unambiguously indicating its typical AIE attributes. Similarly, an enhanced fluorescence of TFMN was also observed when the water fraction in mixed solvent surpassed 75%, and the fluorescence intensity maximum was obtained upon 90% of water fraction showing only 2.6‐fold intensity increase than that of pure ACN solution (Figure S9, Supporting Information). The superb AIE property of TTFMN was also verified by the much higher quantum yield values in aggregate state (*Ф*_F, aggr._ = 4.3%) and solid state (*Ф*_F, solid_ = 16.9%) than that of TFMN (*Ф*_F, aggr._ = 2.7%, *Ф*_F, solid_ = 3.2%) (Figure 2E). It was noteworthy that the emission maxima of TFMN and TTFMN both fell in the NIR region (>650 nm) in solid state (Figure 2D), implying the great potential for NIR fluorescence bioimaging application. Moreover, the lifetimes of TFMN and TTFMN were determined to be 1.64 and 2.52 ns, respectively (Figure 2F).

### 2.3 ROS Generation Evaluation and Theoretical Explanation

**Figure 3:**
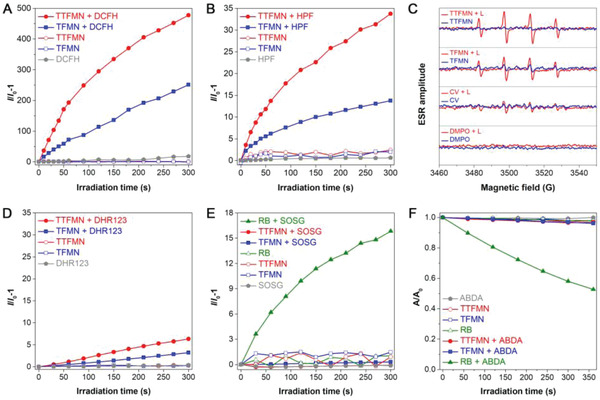
ROS generation of TFMN and TTFMN upon white light irradiation. Relative changes in PL intensity of A) DCFH (for overall ROS detection) and B) HPF (for ^•^OH detection) in the presence of TFMN or TTFMN (2 × 10^−6^ m) upon white light irradiation (22.1 mW cm^−2^) for different times. C) ESR signals of DMPO for type‐I ROS characterization in the presence of TFMN or TTFMN (1 × 10^−3^ m) before and after white light irradiation (200 mW cm^−2^). Relative changes in PL intensity of D) DHR123 (for **^•^** O_2_^−^ detection), E) SOSG (for ^1^O_2_ detection), and F) decomposition rates of ABDA (for ^1^O_2_ detection) in the presence of TFMN or TTFMN (2 × 10^−6^ m) upon white light irradiation (22.1 mW cm^−2^) for different times.

To evaluate the photosensitive abilities of TFMN and TTFMN, their overall ROS production efficiencies were investigated by adopting a classic ROS indicator dichlorofluorescein (DCFH), whose green fluorescence can be sensitively activated by any type of ROS. As shown in **Figure** **3A**, along with the continuous irradiation of white light, the fluorescence intensity of DCFH enhanced rapidly in the presence of TFMN or TTFMN, while negligible increase in fluorescence signal was detected for the irradiated solution with AIEgen or DCFH alone (Figure S10, Supporting Information). Notably, in the presence of TTFMN, the emission intensity of DCFH rose to nearly 500‐fold higher than the initial intensity after 5 min white light irradiation, exhibiting much superior overall ROS production efficiency than TFMN (251‐fold). This result matched well with the calculated smaller Δ*E*_s–t_ of TTFMN (0.20 eV) than that of TFMN (0.24 eV) (Figure S8, Supporting Information). In addition, TTFMN showed comparable overall ROS generation capacity in comparison to the popular commercially available PSs, such as Rose Bengal (RB) and Chlorin e6 (Ce6) (Figure S11, Supporting Information).

The ROS type was further distinguished by employing different ROS indicators.^[^ ^43^, ^44^ ^]^ Initially, the **^•^** OH generation was inspected using hydroxyphenyl fluorescein (HPF) as indicator, which can emit green fluorescence centered at 515 nm upon reaction with **^•^** OH. Meanwhile, crystal violet (CV) as a previously reported type‐I PS was selected as the reference.^[^ ^45^ ^]^ As shown in Figure 3B, AIEgen or HPF alone was almost nonemissive during 5 min white light irradiation. However, the fluorescence intensity of HPF enhanced obviously with the increasing of irradiation time in the presence of TFMN or TTFMN, especially for TTFMN, suggesting that both TFMN and TTFMN were capable of generating **^•^** OH effectively through type‐I process. By contrast, only slight fluorescence enhancement was observed in the presence of CV under the same conditions, further indicating the superior **^•^** OH generation abilities of TFMN and TTFMN (Figure S12, Supporting Information). To further confirm the **^•^** OH production, electron spin resonance (ESR) measurement was carried out using 5,5‐dimethyl‐1‐pyrroline‐*N*‐oxide (DMPO) as the spin‐trap agent (Figure 3C). As expected, typical ESR spectra of characteristic paramagnetic adducts formed with DMPO were observed in the presence of AIEgen after light irradiation for only 1 min. According to the literature,^[^ ^46^ ^]^ these four‐line ESR signals with intensity ratio of about 1:2:2:1 could be attributed to DMPO—**^•^** OH, indicating the distinct production of **^•^** OH. Moreover, TTFMN showed the strongest ESR signal intensity in comparison with TFMN and CV, manifesting its best generation ability of **^•^** OH, consistent with the results detected by HPF. Furthermore, we utilized dihydrorhodamine 123 (DHR123) as indicator to test type‐I ROS ^•^O_2_^−^, and indicators of 9,10‐anthracenediyl‐bis(methylene) dimalonic acid (ABDA) and singlet oxygen sensor green (SOSG) to detect type‐II ROS ^1^O_2_ (Figure 3D–F). The results showed that only slight fluorescence enhancement of DHR123, and nearly no apparent signal change of ABDA or SOSG were found in the presence of TFMN or TTFMN upon white light irradiation, implying their infirm **^•^** O_2_^−^ generation and poor ^1^O_2_ production capacities. Taken together, it was reasonable to conclude that both of these two AIEgens could efficiently generate type‐I ROS **^•^** OH, while TTFMN indeed outperformed TFMN.

On the basis of the well‐recognized electron transfer mechanism of type‐I ROS generation, it was proposed that the triplet TFMN or TTFMN should be able to transfer electrons to the surrounding O_2_. Herein, in order to theoretically validate its feasibility, we calculated the Gibbs free energy changes of this electron transfer process by using Weller equation,^[^ ^47^, ^48^ ^]^ which were calculated to be −0.218 and −0.359 eV for TFMN and TTFMN, respectively. These results solidly revealed that the electron transfer process from TFMN or TTFMN to O_2_ is thermodynamically allowed, indicating that TFMN or TTFMN could transfer electrons to O_2_ and subsequently yield type‐I ROS. Moreover, the more negative Gibbs free energy change of TTFMN (−0.358 eV) than that of TFMN (−0.218 eV) means the larger electron transfer driving force between TTFMN and O_2_, which could be responsible for the higher type‐I ROS generation efficiency of TTFMN. In addition, we further verified that TFMN or TTFMN could first take one electron from the environmental hydroxyl anion and turn into intermediate radical anions (**^•^** PS**^−^**) before they transfer electron to O_2_, with the Gibbs free energy changes of −73.21 and −90.07 kcal mol^−1^ for TFMN and TTFMN calculated by PWPB95‐D3/def2‐TZVP using the quantum mechanism package ORCA 4.1.1 (Figure S14, Supporting Information).^[^ ^49^, ^50^ ^]^ Similarly, the more negative value of TTFMN accounted for its better type‐I ROS generation performance. Consequently, it is reasonable to conclude that the type‐I ROS generation process of TFMN and TTFMN is consistent with the previously reported mechanism,^[^ ^7^, ^51^ ^]^ meaning that triplet TFMN or TTFMN first takes one electron from the environmental hydroxyl anion, and subsequently transfers the electron to O_2_, accompanying with the generation of ^•^O_2_^−^, which could be further transformed into ^•^OH via secondary reactions ultimately (Figure 1).

### 2.4 Design, Preparation, and Characterization of Nuclear Targeting Nanoparticles

Inspired by the hypersensitivity of nucleus to PDT and higher oxidative activity of **^•^** OH to DNA and RNA, in order to take maximum advantage of the type‐I‐**^•^** OH‐mediated PDT based on TTFMN, a nuclear targeting nanodelivery system was tactfully constructed by employing acid‐activated TAT‐functionalized amphiphilic copolymers as encapsulation matrixes. TAT is a type of nuclear localization signal peptide that has been recognized to be effective for translocating NPs and other cargos into cell nuclei.^[^ ^52^, ^53^, ^54^ ^]^ Aiming to inhibit the nonspecific interactions of TAT with blood components and prolong the NP blood retention time, primary amine residues of lysine in TAT that act as the leading cause of nonspecificity but also play a vital role in nuclear localization were skillfully modified with succinic anhydride (SA) to mask the TATʹ activity, moreover, the yielded succinyl amides in TAT could also quickly hydrolyze in specific pH condition corresponding to endo‐/lysosomal acidity (pH 4–5) to fully recover the TATʹ membrane‐transduction and nucleus‐locating functions.^[^ ^55^, ^56^ ^]^ The SA‐modified TAT (TAT^SA^) was further decorated to the terminal of maleimide‐end capped poly(ethylene glycol)‐*block*‐poly(lactic acid) (PLA12k–PEG5k–Mal) to obtain the desired amphiphilic copolymers, namely PLA12k–PEG5k–TAT^SA^. The ultimate nucleus‐targeted nanocarriers were acquired via coassembly of PLA12k–PEG5k–TAT^SA^ and PLA12k–PEG5k, in which the incorporation of PLA12k–PEG5k was expected to enhance the colloidal stability and optimize the particle size of the as‐prepared NPs. After optimizations, the nanodelivery systems containing 85 mol% PLA12k–PEG5k and 15 mol% PLA12k–PEG5k–TAT^SA^ were utilized to encapsulate the TTFMN molecules through a nanoprecipitation method to afford the TTFMN‐loaded NPs (abbreviated as TTFMN‐NPs) for subsequent application studies.

**Figure 4:**
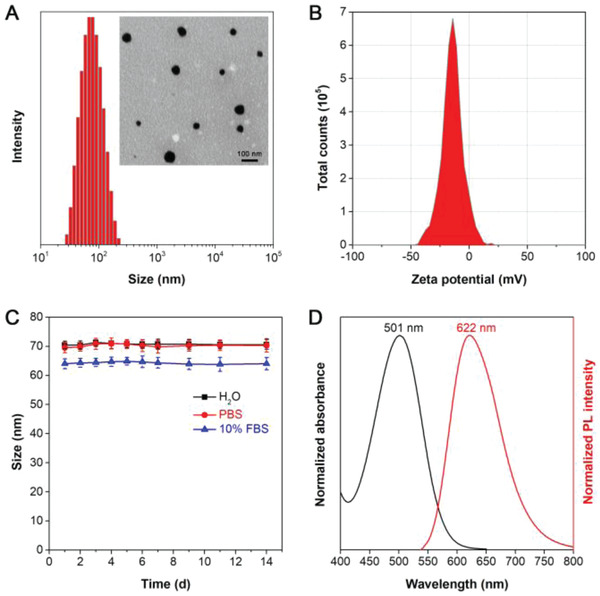
Design and characterization of TTFMN‐NPs. A) DLS profile and TEM image (inset) of TTFMN‐NPs at pH 7.4. B) Zeta potential of TTFMN‐NPs characterized by DLS in aqueous solution at pH = 7.4. C) Stability analysis for size variations of TTFMN‐NPs with a concentration of 100 µg mL^−1^ as a function of storage time at room temperature in H_2_O, PBS, or PBS + 10% FBS measured by DLS. D) Normalized absorption and emission spectra of TTFMN‐NPs in aqueous solutions.

Dynamic light scattering (DLS) measurement revealed the TTFMN‐NPs had an average hydrodynamic diameter of 70 nm in water, which was within the optimal particle size range for passive accumulation into tumors via the enhanced permeability and retention effect (**Figure** **4A**). The representative transmission electron microscope (TEM) image indicated that TTFMN‐NPs possessed spherical nanostructures with the diameter ranging from 30 to 60 nm. Zeta potential of TTFMN‐NPs was further monitored to be −14.5 mV at pH 7.4 (Figure 4B). In addition, the particle size of TTFMN‐NPs remained almost unchanged over 14 days storage in either phosphate buffer saline (PBS) or 10% serum‐contained PBS, manifesting the excellent long‐term colloidal stability which was conducive to their blood circulation capacity (Figure 4C). Afterward, the absorption and emission spectra of TTFMN‐NPs were measured with the corresponding peaks at 501 and 622 nm, respectively (Figure 4D).

### 2.5 Cellular Internalization, pH‐Triggered Lysosomal Escape, and Nuclear Targeting

**Figure 5:**
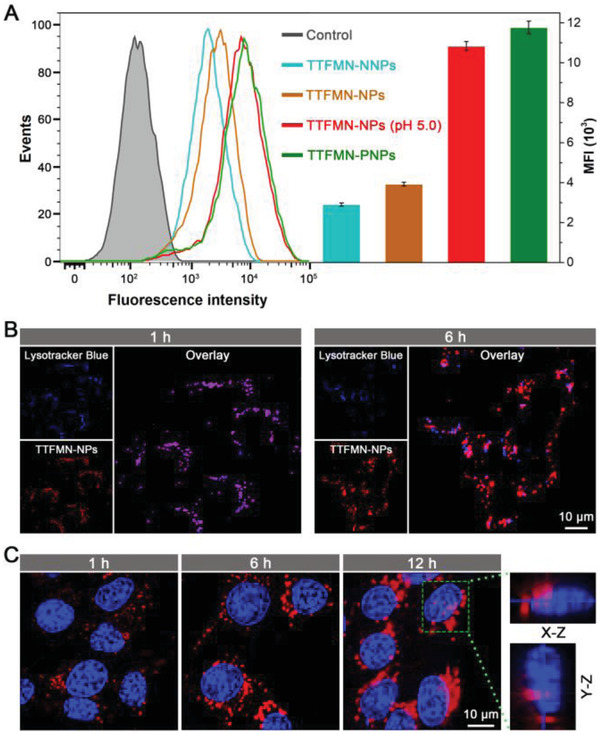
Cellular internalization and intracellular localization of TTFMN‐NPs on 4T1 tumor cells. A) TTFMN‐positive cells and MFI for quantitative cellular uptake after incubation with TTFMN‐NNPs, TTFMN‐NPs, TTFMN‐NPs (pretreated at pH 5.0 for 24 h), and TTFMN‐PNPs for 3 h at a unified TTFMN dose of 10 µg mL^−1^ measured by flow cytometry. B) Intracellular tracking of TTFMN‐NPs (2 µg mL^−1^ TTFMN) on 4T1 cells after incubation for 1 and 6 h imaged by CLSM. C) Nuclear targeting delivery of TTFMN‐NPs (2 µg mL^−1^ TTFMN) on 4T1 cells after incubation for 1, 6, and 12 h determined by CLSM.

Subsequently, the pH‐activated TAT function of TTFMN‐NPs was investigated at cell and tumor sphere levels. As negative and positive controls, TTFMN‐loaded PLA12k–PEG5k micelles (defined as TTFMN‐NNPs) and TTFMN‐loaded hybrid micelles (defined as TTFMN‐PNPs) coassembled from 85 mol% PLA12k–PEG5k and 15 mol% PLA12k–PEG5k–TAT (without SA shelter) were also fabricated. It was observed that TTFMN‐NNPs and unactivated TTFMN‐NPs only induced a weak red fluorescence signals in 4T1 cells after 3 h incubation (**Figure** **5A**). Attractively, the activated TTFMN‐NPs pretreated at pH 5.0 provided markedly enhanced ability to enter the 4T1 cells with about 10.8 × 10^3^ mean fluorescence intensity (MFI) value, which was much better than negative control of TTFMN‐NNPs (2.9 × 10^3^ MFI value) and unactivated TTFMN‐NPs (3.9 × 10^3^ MFI value). Encouragingly, the cellular uptake efficiency of the preactivated TTFMN‐NPs was comparable to that of positive control of TTFMN‐PNPs (11.8 × 10^3^ MFI value), implying the full recovery of TAT activity on the TTFMN‐NP surface. Moreover, multicellular tumor spheroids were constructed as 3D in vitro tumor models to assess the pH‐activated tumor penetrating performance of TTFMN‐NPs (Figure S15, Supporting Information). As expected, activated TTFMN‐NPs exhibited much superior penetration ability, offering strong and homogeneous red fluorescence signals even in the equatorial section of the spheroids, while only a slight red fluorescence was found on the rim of the tumor spheroids incubated with unactivated TTFMN‐NPs. These results clearly indicated that the shielded TAT capabilities of membrane‐transduction and tumor‐penetrating in TTFMN‐NPs could be successfully activated at specific pH condition corresponding to endo‐/lysosomal acidity.

Motivated by the acid‐activated TAT functions, we immediately tracked the intracellular delivery and distribution of TTFMN‐NPs on 4T1 cells. As depicted in Figure 5B and Figure S16 (Supporting Information), TTFMN‐NPs were initially located in the endo‐/lysosomes with a short incubation time of 1 h, confirmed by the excellent colocalization of TTFMN‐NPs with endo‐/lysosomes. After extending the incubation period to 6 h, a distinct escape of TTFMN‐NPs from the endo‐/lysosomes was clearly observed, as evidenced by the dramatically decreased Pearson's correlation coefficient (0.59) between red and blue fluorescence, convincingly demonstrating that the endo‐/lysosomal acidity was able to efficiently activate the membrane‐penetrating features of TAT on the surface of TTFMN‐NPs. Next, the nuclear targeting activity of TTFMN‐NPs was assessed (Figure 5C). Consistent with the aforementioned results, few TTFMN‐NPs were found in the cell nuclei within a short incubation time of 1 h. Prolonging the incubation time to 6 h made more TTFMN‐NPs enter the 4T1 cells, some of them transfer to the perinuclear regions. Once the incubation time was extended to 12 h, a considerable amount of TTFMN‐NPs were found to be internalized into the cells, traverse to the perinuclear regions, punctuate on the nuclear membranes, and even partially enter into the nuclei. The nucleus‐anchoring ability of TTFMN‐NPs could be further clearly manifested by the enlarged z‐stacking images of one cell nucleus. It is worth mentioning that owing to the large average particle size (70 nm) of TTFMN‐NPs, the majority of the NPs were docked on the nuclear pores, while only a small amount could enter the nuclei. These results in the current work supported the previous findings that TAT could ship particles with diameters smaller than 50 nm freely through the nuclear pores, but large particles (>67 nm) could not.^[^ ^57^ ^]^ Collectively, TTFMN‐NPs were internalized into the 4T1 cells via endocytic pathway, and the subsequent acidic environments of endo‐/lysosomes could efficiently trigger their membrane‐penetrating and nucleus‐locating properties to implement successful lysosomal escape and nuclear targeting delivery. Additionally, photostability evaluation measurement indicated that TTFMN‐NPs exhibited high photobleaching resistant, which could significantly favor the fluorescence imaging‐guided photodynamic therapy (Figure S17, Supporting Information).

### 2.6 Photodynamic Tumoricidal Effect Study In Vitro

**Figure 6:**
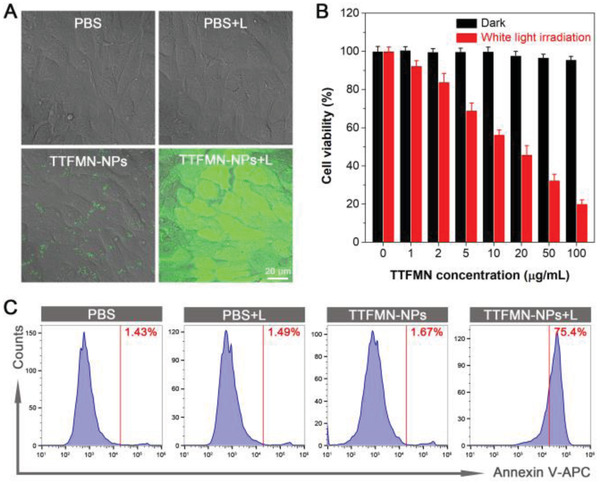
Photodynamic tumoricidal effect of TTFMN‐NPs on 4T1 tumor cell. A) Intracellular ROS level of 4T1 cells after various treatments indicated by DCFH‐DA. Laser irradiation (488 nm, 2% power, 3 min) was conducted after cells were incubated with TTFMN‐NPs (50 µg mL^−1^ TTFMN) for 24 h. B) Cell viability of 4T1 cells incubated with TTFMN‐NPs at various concentrations in the dark and after white light irradiation (50 mW cm^−2^) determined by MTT assay (mean ± SD, *n* = 6). C) Apoptosis analysis using flow cytometry toward 4T1 cells after different treatments. White light irradiation (50 mW cm^−2^) was conducted after cells were incubated with TTFMN‐NPs (50 µg mL^−1^ TTFMN).

In light of the splendid type‐I ROS generation and precise nuclear targeting delivery of TTFMN‐NPs, the light‐induced tumoricidal activities of TTFMN‐NPs toward 4T1 cells were estimated. Initially, the light‐triggered ROS production of TTFMN‐NPs inside cells was studied upon exposure to a 488 nm laser equipped in the confocal laser scanning microscope (CLSM) (**Figure** **6A** and Figure S18 (Supporting Information)). Within a short irradiation period of 3 min, green DCFH fluorescence signal was distinctly observed in the cells, revealing that TTFMN‐NPs with light irradiation could efficiently arouse intracellular ROS generation. In the following text, photodynamic antitumor effect of TTFMN‐NPs to 4T1 cells was evaluated using 3‐(4,5‐dimethyl‐2‐thiazolyl)‐2,5‐diphenyltetrazolium bromide (MTT) assay. As shown in Figure 6B and Figure S19 (Supporting Information), TTFMN‐NPs exhibited negligible dark cytotoxicity to 4T1 tumor cells as well as 3T3 and human umbilical vein endothelial ​cells (HUVEC) normal cells even at high concentration, manifesting their excellent biocompatibility. Upon the introduction of white light irradiation, a dose‐dependent phototoxicity against 4T1 cells was clearly observed, implying the salient photodynamic tumoricidal efficacy of TTFMN‐NPs. In order to reveal the phototherapeutic effect of TTFMN‐NPs intuitively, fluorescein diacetate (FDA) and propidium iodide (PI) costaining assay was carried out to distinguish the live and dead 4T1 cells (Figure S20, Supporting Information). Consistent with the MTT assay results, a great amount of 4T1 cells were PI‐positive and presented death state after treatment with TTFMN‐NPs plus white light irradiation, demonstrating the effectiveness and controllability of PDT. Moreover, aiming to get further insights into the phototherapeutic mechanism of TTFMN‐NPs, 4T1 cells previously receiving different treatments were labeled with anti‐*γ*‐H2AX by using immunofluorescence cell staining method. As shown in Figure S21 (Supporting Information), compared to the cells in the control groups, stronger red fluorescent signal was observed in the nuclei of 4T1 cells treated with both TTFMN‐NPs and white light irradiation. Since *γ*‐H2AX, the phosphorylated form of histone protein H2AX, represents an efficient biomarker for DNA double‐strand breaks,^[^ ^58^ ^]^ these results definitely indicated that TTFMN‐NPs could induce DNA damage through generating type‐I ROS upon white light irradiation. In addition, the apoptosis assay was carried out by using Annexin V–APC (Allophycocyanin) staining to validate the type‐I‐PDT‐induced cell apoptosis by flow cytometry analysis (Figure 6C). It turned out that a significantly high proportion (about 75.4%) of cells presented APC‐positive after treatment with TTFMN‐NPs plus white light irradiation, meaning that TTFMN‐NPs with light irradiation induced the 4T1 tumor cell death mainly through type‐I‐PDT‐mediated apoptotic pathway. Evidently, by making the best of the combined superiorities of generated type‐I ROS and nucleus‐targeted delivery, TTFMN‐NPs smoothly brought about severe tumor cell apoptosis triggered by white light irradiation.

### 2.7 In Vivo Fluorescence‐Imaging‐Guided Photodynamic Therapeutic Efficiency of TTFMN‐NPs

**Figure 7:**
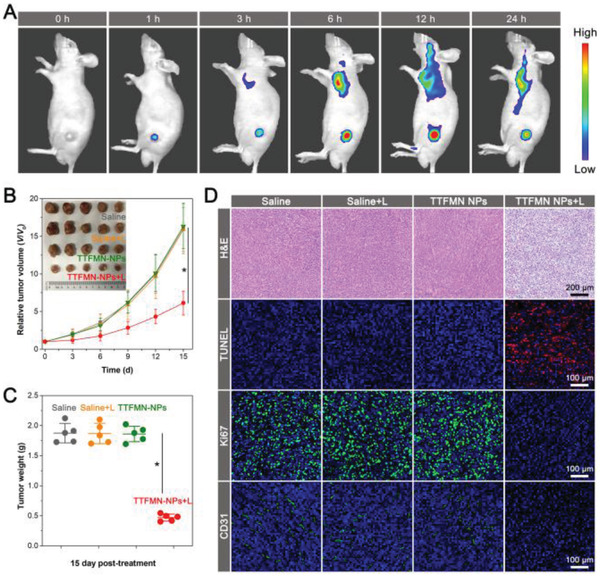
In vivo fluorescence‐imaging‐guided photodynamic therapeutic efficiency of TTFMN‐NPs on 4T1‐tumor‐bearing BALB/c nude mice through systemic administration. A) Fluorescence images of tumor‐bearing mice at different monitoring times after administration of TTFMN‐NPs. B) Time‐dependent tumor growth curves of tumor‐bearing mice with various treatments (*n* = 5, *p < 0.001). Inset: photos of the tumors harvested at day 15 after different treatments. C) Weights of the tumors harvested at day 15 after different treatments (*n* = 5, *p < 0.001). D) H&E, TUNEL, Ki67, and CD31 staining analysis of tumor tissues after various treatments. TUNEL, Ki67, and CD31‐positive cells were stained red, green, and green, respectively.

The performance of TTFMN‐NPs for in vivo NIR fluorescence imaging was assessed on the 4T1‐tumor‐bearing BALB/c nude mice. After intravenous injection of TTFMN‐NPs for a short time of 1 h, obvious NIR fluorescence was detected at the tumor site, indicating the quick accumulation into tumor tissues. With the extension of observation time, the fluorescence intensity in tumor location increased gradually and reached a maximum at 12 h postinjection, suggesting 12 h would be the optimal time point for the subsequent PDT. Afterward, a fluorescence reduction attributing to the normal physiological metabolism was subsequently observed. Even so, the fluorescence signals of TTFMN‐NPs at tumor site were still distinctly detectable after 24 h injection, showing their relative strong retention ability in tumor (**Figure** **7A** and Figure S22 (Supporting Information)). The ex vivo imaging results indicated that tumor tissue exhibited significantly stronger fluorescence signal than any other organs, indicating the effectively preferential accumulation of TTFMN‐NPs in tumor (Figure S23, Supporting Information). Subsequently, the in vivo phototherapeutic study was conducted. The tumor growth curves indicated that the tumor volumes in control groups increased sharply during the study period and no significant difference was observed among them. Remarkably, upon the introduction of white light irradiation, TTFMN‐NPs exhibited obvious tumor growth inhibition effect (Figure 7B). The weight of dissected tumors after 15 days complete treatment further demonstrated the highly effective PDT efficacy of TTFMN‐NPs on the tumor‐bearing mice with a tumor inhibition rate as high as 75.1% (Figure 7C). Additionally, the hematoxylin and eosin (H&E) staining results of the dissected tumors, exhibiting vacuolization, massive nucleus absence, and conspicuous karyopyknosis, suggested a successful destruction of tumor tissue caused by PDT (Figure 7D). Terminal‐deoxynucleotidyl transferase‐mediated nick end labeling (TUNEL) staining assay further verified the severe cell apoptosis of tumor tissue induced by TTFMN‐NPs with white light irradiation, in which a mass of TUNEL‐positive red fluorescence signals could be observed. The therapeutic mechanism was also investigated by monoclonal antibody Ki67 and platelet endothelial cell adhesion molecule‐1 (CD31) immunofluorescence staining assays. Supporting the H&E and TUNEL staining results, few Ki67‐positive proliferating cells and CD31‐positive new microvessels were found in the TTFMN‐NPs plus light irradiation group. In sharp contrast, densely arranged tumor cells with extremely strong proliferation activity and abundant neovessels appeared in the tumor tissues of control groups. These results convincingly demonstrated that nucleus‐targeted delivery associated with type‐I PDT damage could effectively inhibit tumor growth by inducing tumor cell apoptosis as well as suppressing tumor cell proliferation and tumor vessel generation.

The potential systemic toxicity or biological safety is always a great concern for theranostic agents used in clinic. Therefore, the in vivo biosecurity of TTFMN‐NPs was carefully evaluated. Primarily, the body weights of all the treated mice were measured every 3 days during the treatment course and reasonable weight changes in normal range were noticed for each group (Figure S24, Supporting Information). In addition, after intravenous injection of TTFMN‐NPs for one week, the healthy mice were sacrificed and the blood was collected for serum biochemical and hematological analyses. Compared with the saline‐injected group, the blood biochemistry parameters of hepatic function markers including alanine aminotransferase (ALT), aspartate transaminase (AST), albumin (ALB), and renal function markers including creatinine (CR), uric acid (UA), blood urea nitrogen (BUN), as well as routine blood indexes in the TTFMN‐NP‐administered group all were presented within normal range and no apparent difference was observed, suggesting the negligible systemic side effects of TTFMN‐NPs (Figure S25 and Table S2, Supporting Information). At the same time, the main organs of the mice were also exfoliated and subjected to histological examination (Figure S26, Supporting Information). As revealed in H&E‐stained slices, all the organs in TTFMN‐NP‐administered mice exhibited normal tissue morphology without any noticeable organ damage or inflammatory lesion observed, offering another solid evidence for TTFMN‐NPs with appreciable biosecurity.

## 3 Conclusion

In conclusion, a nuclear targeting PDT system based on type‐I AIE PSs was tactfully designed and successfully constructed in this work. Two AIE‐active PSs TFMN and TTFMN with long‐emission wavelength were facilely synthesized and demonstrated to exhibit high **^•^** OH generation efficiency, presumably ascribing to the high electron affinity of the cyano groups. Benefiting from the more twisted and propeller‐like conformation as well as smaller calculated Δ*E*_s–t_, TTFMN showed superior both AIE tendency and **^•^** OH production capability to TFMN. A lysosomal acid‐activated TAT‐peptide‐modified amphiphilic polymer PLA12k–PEG5k–TAT^SA^ was further employed to transport well‐tailored TTFMN precisely into cell nuclei in order to maximize the PDT efficiency involving type‐I ROS. In vitro and in vivo investigations illustrated that those fabricated TTFMN‐NPs sharing appreciable biocompatibility and fairly favorable blood circulation capacity were able to specifically accumulate at tumor site and undergo efficient cellular uptake through lysosome‐mediated endocytosis pathway. Subsequently, the nuclear targeting activity of TAT on the surface of TTFMN‐NPs was successfully recovered in the lysosomal acidic condition, thus potentiating the efficient nuclear accumulation of TTFMN and ROS generation in the nucleus region upon white light irradiation. These distinct features endowed TTFMN‐NPs with excellent performance in NIR‐fluorescence‐imaging‐guided nucleus‐targeted PDT applications. These findings in this study would open up new perspectives in the design of advanced PSs, and trigger state‐of‐the‐art developments of type‐I PDT involving nuclear targeting delivery in potential clinical trials.

## 4 Experimental Section

### Materials

Amphiphilic copolymers PLA12k–PEG5k and PLA12k–PEG5k–TAT^SA^ were customized from Xi'an ruixi Biological Technology Co., Ltd. LysoTracker Blue was purchased from KeyGEN BioTECH Co., Ltd. 2ʹ,7ʹ‐dichlorodihydrofluorescein diacetate (DCFH‐DA), HPF, ABDA, FDA, PI, RB, and MTT were purchased from Sigma‐Aldrich. Ce6 was obtained from J&K. DHR123 and CV were offered from Aladdin Co., Ltd. SOSG and PBS were purchased from Thermo Fisher Scientific. DMPO, Hoechst 33342, and Apoptosis Assay Kit (Annexin V–APC) were purchased from Dojindo Laboratories. Monoclonal antibody against *γ*‐H2AX and Alexa Fluor 647‐labeled goat anti‐rabbit secondary antibody were purchased from Cell Signaling Technology, Inc. (USA). Roswell Park Memorial Institute‐1640 medium, fetal bovine serum (FBS), penicillin, and streptomycin were purchased from Gibco. 1‐(4‐Bromophenyl)‐1,2,2‐triphenylethylene, aniline, 1‐bromo‐4‐iodobenzene, 4‐bromo‐*N*,*N*‐diphenylaniline, (5‐formylfuran‐2‐yl)boronic acid, malononitrile, Pd(dppf)Cl_2_, tri‐*tert*‐butylphosphine, sodium *tert*‐butoxide, 1,10‐phenanthroline, CuI, KOH were purchased from J&K or Meryer. All the chemicals were used as supplied without further purification. Tetrahydrofuran (THF) was dried by distillation using sodium as drying agent and benzophenone as indicator.

### Characterization

^1^H and ^13^C NMR spectra were recorded on a Bruker ARX 400 NMR spectrometer. High‐resolution mass spectra (HRMS) were obtained on a Finnigan MAT TSQ 7000 Mass Spectrometer operating in a matrix‐assisted laser desorption/Ionization time‐of‐flight (MALDI‐TOF) mode. Quantum yield was determined by a Quanta‐integrating sphere. ESR analysis was performed on a Bruker EMS^plus^‐10/12 spectrometer. Absorption spectra were measured on a PerkinElmer Lambda 950 spectrophotometer. PL spectra were recorded on Edinburgh FS5 fluorescence spectrophotometer. Size distribution and zeta potential were analyzed on a DLS using a Malvern Zetasizer Nano ZSP. Particle size and morphology were observed on a HITACHI‐HT7700 transmission electron microscope. Laser confocal scanning microscope images were collected on a confocal laser scanning microscope (CLSM, ZEISS‐LSM880). The cell viability was detected by MTT assay, and the absorbance of each sample was measured at 570 nm using a microplate reader (BioTek). The cell internalization efficiency and apoptosis analysis were evaluated on a BD FACSAria SORP fluorescence activated cell sorting.

### Synthesis of Compound **1**

In the presence of Pd(dppf)Cl_2_ (73.1mg, 0.1 mmol) and K_2_CO_3_ (414.6 mg, 3.0 mmol), a mixture of 4‐bromo‐*N*,*N*‐diphenylaniline (324.2 mg, 1.0 mmol), (5‐formylfuran‐2‐yl)boronic acid (279.8 mg, 2.0 mmol) were dissolved in mixed solvent (MeOH:toluene = 5:5 mL). The reaction was heated to 75 °C for 16 h under nitrogen. After the reaction was complete, the mixture was cooled to room temperature and filtered, from which the solvent was removed by reduced pressure. Subsequently, the residue was dissolved in dichloromethane (100 mL) and washed by water (50 mL × 3). The combined organic phase was dried over Na_2_SO_4_, filtered, and concentrated to obtain the crude product. Further purification by silica gel chromatography gave the desired product with a yield of 72.0% (244.4 mg).

### Synthesis of TFMN

Synthetic procedures toward TFMN was referred to a literature reported method.^[^ ^59^ ^]^ To a solution of compound **1** (339.4 mg, 1.0 mmol) in EtOH, malononitrile (132.1 mg, 2.0 mmol) was added. The mixture was refluxed for 72 h, followed by solvent removal. The residue was purified by silica gel chromatography to generate the product with a yield of 88.0% (340.9 mg). ^1^H NMR (400 MHz, chloroform‐d) *δ* 7.68 (d, *J* = 8.8 Hz, 2H), 7.35–7.28 (m, 5H), 7.25–7.2 (m, 1H), 7.18–7.1 (m, 6H), 7.10–7.04 (m, 2H), 6.81 (d, *J* = 3.9 Hz, 1H). ^13^C NMR (100 MHz, chloroform‐d) *δ* 162.27, 150.32, 146.93, 146.66, 140.54, 129.74, 127.09, 125.84, 124.62, 121.42, 120.61, 115.10, 114.06, 108.42, 73.38. MALDI‐HRMS: *m*/*z* calcd. for C_26_H_17_N_3_O 387.1372, found 387.1364.

### Synthesis of Compound **2**

Intermediate compound **2** was synthesized according to the literature method.^[^ ^60^ ^]^ 1‐(4‐Bromophenyl)‐1,2,2‐triphenylethylene (205.0 mg, 0.5 mmol), aniline (55.8 mg, 0.6 mmol), tri‐*tert*‐butylphosphine (1.6 mg, 0.008 mmol), Pd_2_(dba)_3_ (6.4 mg, 0.007 mmol), and sodium *tert*‐butoxide (57.6 mg, 0.6 mmol) were mixed with dry toluene (10 mL) in a two‐necked round bottom flask containing a stir bar. The mixture was stirred at 120 °C under N_2_ atmosphere for 24 h. After solvent removal, water (30 mL) and chloroform (100 mL) were added. The organic layer was separated and washed with brine, dried over anhydrous MgSO_4_, and evaporated to dryness under reduced pressure. The crude product was purified by column chromatography on silica gel using hexane/chloroform (v/v = 20/1) as eluent to afford compound **2** as pale yellow solids in 78.5% yield (162.1 mg).

### Synthesis of Compound **3**

A mixture of compound *N*‐phenyl‐4‐(1,2,2‐triphenylvinyl)aniline (**2**) (296.1 mg, 0.7 mmol), 1‐bromo‐4‐iodobenzene (338.2 mg, 1.2 mmol), 1,10‐phenanthroline, (216.2 mg, 1.2 mmol), CuI (228.5 mg, 1.2 mmol), KOH (117.8 mg, 2.1 mmol), and anhydrous toluene (50 mL) was stirred at 120 °C under N_2_ atmosphere for 48 h. After the reaction was completed, the mixture was cooled down and the solvent was removed by a rotary evaporator. The crude product was redissolved in dichloromethane and then washed with water for 3 times. Solvent was removed and the resultant crude product was purified by silica column with hexane/dichloromethane (v/v = 50/1) as the eluent to afford compound **3** as a light yellow solid (143.8 mg, yield 35.1%).

### Synthesis of Compound **4**

The synthetic process was similar to compound **1** except for the change of starting materials. Further purification by silica gel chromatography gave the desired product with a yield of 54.7% (210 mg).

### Synthesis of TTFMN

To a solution of compound **4** (140.1 mg, 0.236 mmol) in EtOH, malononitrile (62.4 mg, 0.943 mmol) was added. The mixture was refluxed for 72 h, followed by solvent removal. The residue was purified by silica gel chromatography to generate the product with a yield of 85.9% (130.1 mg). ^1^H NMR (400 MHz, chloroform‐d) *δ* 7.67 (d, *J* = 8.8 Hz, 2H), 7.27–7.33 (m, 4H), 7.0–7.16 (m, 20 H), 6.94 (d, *J* = 8.4 Hz, 2H), 6.85 (d, *J* = 8.8 Hz, 2H), 6.8 (d, *J* = 4.0 Hz, 1H). ^13^C NMR (100 MHz, chloroform‐d) *δ* 162.08, 149.91, 146.76, 146.28, 144.56, 143.81, 143.50, 143.26, 141.12, 140.35, 139.90, 132.46, 131.33, 131.30, 131.28, 129.48, 127.71, 127.62, 127.60, 126.86, 126.53, 126.43, 125.56, 124.46, 124.38, 121.42, 120.47, 114.96, 113.93, 108.27, 73.15. MALDI‐HRMS: calcd. for C_46_H_31_N_3_O [M]^+^: 641.2467, found: 641.2463.

### Overall ROS Detection in Aqueous Solution

A commonly used ROS indicator DCFH‐DA was utilized to detect the ROS generation of AIEgens in aqueous solution under white light irradiation (22.1 mW cm^−2^). DCFH‐DA was prehydrolyzed into DCFH according to the reference.^[^ ^33^ ^]^ Then, the ROS indicator (40 × 10^−6^ m) in PBS was further diluted to 5 × 10^−6^ m in the sample solution of AIEgens, RB, or Ce6 (2 × 10^−6^ m) for measurement by PL instrument. The fluorescence of 2ʹ,7ʹ‐dichlorofluorescein triggered by AIEgen‐sensitized ROS under white light irradiation was measured at different time intervals. The PL spectra were measured with excitation at 488 nm and emission was collected from 500 to 620 nm. The fluorescence intensity at 525 nm was recorded to indicate the generation rate of overall ROS.

### •OH Detection in Aqueous Solution

The ^•^OH generation potency was evaluated by using HPF as an indicator. The stock solution of HPF (5 × 10^−3^ m in *N,N*‐dimethylformamide) was diluted to 5 × 10^−6^ m in the sample solution of AIEgens (2 × 10^−6^ m) and CV (2 × 10^−6^ m) in PBS buffer. The fluorescence signal of HPF was monitored at different time intervals in a range of 500–620 nm with the excitation wavelength at 490 nm after the solution was irradiated by white light irradiation (22.1 mW cm^−2^). The fluorescence intensity at 515 nm was recorded to indicate the generation rate of ^•^OH.

### ^•^O_2_^−^ Detection in Aqueous Solution

The ^•^O_2_^−^ generation measurements were performed using DHR123 as an indicator. The stock solution of DHR123 (5 × 10^−3^ m) was diluted to 5 × 10^−6^ m in the sample solution of AIEgens (2 × 10^−6^ m) in PBS buffer. The fluorescence signal of DHR123 was monitored at different time intervals in a range of 500–620 nm with the excitation wavelength at 495 nm after the solution was irradiated by white light irradiation (22.1 mW cm^−2^). The fluorescence intensity at 526 nm was recorded to indicate the generation rate of ^•^O_2_^−^.

### ^1^O_2_ Detection in Aqueous Solution

The ^1^O_2_ generation was first assessed by employing SOSG as an indicator. The stock solution of SOSG (5 × 10^−3^ m) was diluted to 5 × 10^−6^ m in the sample solution of AIEgens (2 × 10^−6^ m) and RB (2 × 10^−6^ m) in PBS buffer. The fluorescence signal of SOSG was monitored at different time intervals in a range of 500–620 nm with the excitation wavelength at 488 nm after the solution was irradiated by white light irradiation (22.1 mW cm^−2^). The fluorescence intensity at 525 nm was recorded to indicate the generation rate of ^1^O_2_. For the ^1^O_2_ detection indicated by ABDA, the stock solution of ABDA (20 × 10^−3^ m) was diluted to 20 × 10^−6^ m in the sample solution of AIEgens (2 × 10^−6^ m) and RB (2 × 10^−6^ m) in PBS buffer. The absorption spectra of ABDA were monitored in a range of 330–450 nm after the solution was irradiated by white light irradiation (22.1 mW cm^−2^). The absorbance decrease of ABDA at 400 nm was recorded to indicate the decomposition rates of ABDA (^1^O_2_ generation rate).

### ESR Analysis

ESR analysis was carried out to confirm the generation of ^•^OH using DMPO as spin‐trap agent. The working samples containing 70.8 × 10^−3^ m DMPO and 1 × 10^−3^ m of TFMN, TTFMN, and CV were injected quantitatively into quartz capillaries, and the spectra of spin was monitored before and after the solution was irradiated by irradiation white light (200 mW cm^−2^) for 1 min.

### Calculation of Free Energy Changes of the Electron Transfer Process

The Gibbs free energy changes of the electron transfer between TFMN or TTFMN and O_2_ were calculated with the Weller equation (Equations (1) and (2))

(1) $$\Delta G_{\mathrm{CS}}^{0}=e\left[E_{\mathrm{OX}}-E_{\mathrm{RED}}\right]-E_{00}+\Delta G_{S}$$

(2) $$\Delta G_{S}=-\frac{e^{2}}{4\pi \varepsilon_{s}\varepsilon_{0}R_{\mathrm{cc}}}-\frac{e^{2}}{8\pi \varepsilon_{0}}\left(\frac{1}{R_{D}}+\frac{1}{R_{A}}\right)\left(\frac{1}{\varepsilon_{\mathrm{REF}}}-\frac{1}{\varepsilon_{S}}\right)$$

where *e* is the electronic charge; *E*_OX_ is the oxidation potential of electron‐donor unit (1.01 V vs saturated calomel electrode (SCE) for TFMN, 0.85 V vs SCE for TTFMN) (Figure S13, Supporting Information), measured according to the literature method;^[^ ^61^ ^]^ *E*_RED_ is the reduction potential of the electron‐acceptor unit (O_2_, −0.78 V vs SCE); *E*_00_ is the approximate energy level of triplet state (1.506 eV for TFMN, 1.501 eV for TTFMN), which was calculated by density functional theory calculations at the B3LYP/6‐311G** level using the Gaussian 09 program; Δ*G*_S_ is the static Coulombic energy, which was estimated from Equation (2); *ε*_S_ is the static dielectric constant of the solvent (H_2_O, 78.5); *ε*_0_ is the permittivity of free space (8.85 × 10^−12^); *R*_CC_ is the center‐to‐center separation distance, estimated as +∞ due to it being an intermolecular distance; *R*_D_ is the radius of the electron donor (8 Å for TFMN, 9.5 Å for TTFMN); *R*_A_ is the radius of the electron acceptor (O_2_, 1.73 Å); ɛ_REF_ is the static dielectric constant of solvent used for electrochemical study (dichloromethane, 8.93). Accordingly, the Gibbs free energy changes of the electron transfer from TFMN or TTFMN to O_2_ were calculated to be −0.218 and −0.359 eV, respectively.

### Preparation of TTFMN Nanoparticles

To prepare TTFMN‐NPs, 1 mg of TTFMN and 20 mg of amphiphilic copolymers (85 mol% PLA12k–PEG5k and 15 mol% PLA12k–PEG5k–TAT^SA^) were first dissolved in 1 mL of THF. Then, the 1 mL of THF solution was added into 9 mL of deionized water followed by sonication with a microtip probe sonicator (XL2000, Misonix Incorporated, NY) at 45% output power for 2 min continuously. Then, the mixtures were transferred into dialysis bag with the molecular weight cut off (MWCO) of 3500 Da and dialyzed against deionized water for 24 h. In order to remove THF completely, the water was replaced by fresh water every 4 h. The final obtained solutions of TTFMN‐NPs were freeze‐dried or concentrated by ultrafiltration before use. The loading content was determined to be 4.4 wt% using a pre‐established calibration absorption curve, and the entrapment efficiency was calculated to be 92.6%. TTFMN‐NNPs and TTFMN‐PNPs were prepared following the similar procedure as above, only utilizing different amphiphilic copolymer matrices.

### Cellular Uptake Assay

Mouse breast cancer 4T1 cells were seeded in six‐well plates at a density of 1 × 10^5^ cells per well and cultured in 1640 culture medium containing 10% FBS and 1% antibiotics (penicillin–streptomycin) for 24 h. Then, the media were replaced by fresh complete media containing TTFMN‐NNPs, TTFMN‐NPs, TTFMN‐NPs (pretreated in pH 5.0 PBS buffer for 24 h), or TTFMN‐PNPs and further incubation for 3 h in 37 °C. The concentration of nanoparticles was 10 µg mL^−1^ determined by TTFMN. After that, the cells were gently washed with PBS for 3 times, and then trypsinized and harvested quickly for flow cytometry analysis. The results were analyzed with FlowJo10 software. All experiments were performed with three parallel samples.

### Penetration of TTFMN‐NPs into Breast 3D In Vitro Tumor Spheroid Model

The breast 3D tumor spheroid model was first established according to the previous reported method.^[^ ^62^ ^]^ When the multicellular tumor spheroids grew to an average size of about 100 µm, they were treated with TTFMN‐NPs (10 µg mL^−1^ determined by TTFMN) at 37 °C for 3 h to assess the acid‐activated tumor penetration ability indicated by the fluorescence intensity inside the tumor spheroids. These NPs were pretreated at pH 7.4 or 5.0 PBS for 24 h at 37 °C, respectively. Afterward, the tumor spheroids were washed with PBS for 3 times and imaged by CLSM with z‐stack scanning from the top to the equatorial plane.

### Lysosomal Escape and Nuclear Targeting Delivery

4T1 cells were seeded at a suitable density in glass bottom dish and cultured for 24 h. For the lysosomal escape observation, the cells were further cultured in fresh complete media containing TTFMN‐NPs (2 µg mL^−1^ determined by TTFMN) for 1 and 6 h at 37 °C. Then, the cells were washed with PBS for 3 times and subsequently stained with LysoTracker Blue (LTB) for 30 min. After that, the samples were washed with PBS and observed under CLSM. Conditions: excitation wavelength: 405 nm for LTB and 488 nm for TTFMN‐NPs; emission filter: 410–500 nm for LTB and 550–750 nm for TTFMN‐NPs. For nuclear targeting delivery study, the cells were further cultured in fresh complete media containing TTFMN‐NPs (2 µg mL^−1^ determined by TTFMN) for 1, 6, and 12 h at 37 °C. After being rinsed with PBS for 3 times, and then stained with Hoechst 33342 for 30 min, the samples were imaged using CLSM. Conditions: excitation wavelength: 405 nm for Hoechst 33342 and 488 nm for TTFMN‐NPs; emission filter: 410–500 nm for Hoechst 33342 and 550–750 nm for TTFMN‐NPs.

### Intracellular ROS Generation

4T1 cells were seeded and cultured in glass bottom dish for 24 h. Then, the cells were further treated in fresh media containing TTFMN‐NPs (50 µg mL^−1^ determined by TTFMN) for 24 h. Afterward, the cells were incubated with 1 mL fresh serum‐free media containing 10 × 10^−6^ m DCFH‐DA at 37 °C for 20 min. After washing, the cells were exposed to 488 nm laser irradiation (2% laser power) for 3 min, followed by CLSM imaging. The cells treated with TTFMN‐NPs without laser irradiation or PBS with/without laser irradiation served as the controls. The CLSM images were captured with excitation at 488 nm and emission was collected from 500 to 550 nm.

### Cytotoxicity Evaluation Induced by PDT

MTT assays were used to evaluate the cytotoxicity of TTFMN‐NPs in the dark and under light irradiation. 4T1 cells were seeded on a 96‐well plates at a density of 5 × 10^3^ cells per well and incubated for 12 h. Then, the cells were incubated with different concentrations of TTFMN‐NPs in fresh media. After 24 h incubation, the cells were exposed to white light irradiation (50 mW cm^−2^) for 10 min. Meanwhile, the TTFMN‐NP‐incubated cells without light irradiation were also conducted for the dark cytotoxicity study. After further incubation for 24 h, the media were removed and washed with PBS for 3 times. Cells were then incubated with fresh serum‐free medium containing 10% MTT for 4 h in the dark. Then, all the media were removed and 150 µL dimethyl sulfoxide (DMSO) was added. Finally, the absorbance of the products was measured at a wavelength of 570 nm by a microplate reader. The results were expressed as the viable percentage of cells after different treatments relative to the control cells without any treatment.

### Live/Dead Cell Staining

First, 4T1 cells were seeded and cultured in glass bottom dish for 24 h, then exposed to different following treatments: 1) PBS; 2) irradiated with white light irradiation (50 mW cm^−2^) for 10 min; 3) incubated with TTFMN‐NPs for 24 h; 4) incubated with TTFMN‐NPs for 24 h and irradiated with white light irradiation (50 mW cm^−2^) for 10 min. The concentration of TTFMN‐NPs was 50 µg mL^−1^ determined by TTFMN. After different treatments, the cells were incubated at 37 °C for another 24 h, then successively stained with PI (60 µg mL^−1^) and FDA (100 µg mL^−1^) in PBS for 10 min. Subsequently, the cells were gently washed and then imaged by CLSM. Conditions: excitation wavelength: 488 nm for FDA and 543 nm for PI; emission filter: 500–550 nm for FDA and 550–650 nm for PI.

### PDT‐Mediated Cell Apoptosis Detection

4T1 cells were seeded and cultured in six‐well plates for 24 h at the density of 1 × 10^5^ cells per well. Then, TTFMN‐NPs (50 µg mL^−1^ determined by TTFMN) were added into the culture media. After 24 h incubation, the cells were washed and replaced with fresh media, followed by white light irradiation (50 mW cm^−2^) for 10 min. The cells treated with TTFMN‐NPs without light irradiation or PBS with/without light irradiation served as the controls. After that, the cells were incubated at 37 °C for another 24 h. Subsequently, the cells were collected by centrifugation at 1000 rpm for 5 min and washed with PBS for 3 times at 4 °C. The samples were then stained with an Annexin V–APC according to manufacturer's instructions and analyzed by flow cytometry analysis with excitation at 633 nm and emission filter at 640–680 nm.

### In Vitro DNA Damage Study

For the *γ*‐H2AX immunofluorescence analysis, the 4T1 cells receiving the same treatments as described in “Live/Dead Cell Staining” study were fixed, punched, and blocked sequentially. Then, the cells were incubated with rabbit‐derived anti‐*γ*‐H2AX monoclonal antibody (1:400), followed by incubation with secondary Alexa 647‐conjugated goat anti‐rabbit antibody (1:500). After further staining with Hoechst 33342, the cells were finally imaged by CLSM.

### Animals and Tumor‐Bearing Mouse Model

All animal experiments were carried out in accordance with the protocols approved by the Administrative Committee on Animal Research in Shenzhen Graduate School, Peking University (SYXK(YUE)2017‐0172). Four week old male BALB/c nude mice were purchased from Beijing Vital River Laboratory Animal Technology. All animals were acclimatized to the animal facility for one week prior to experimentation and housed under pathogen‐free conditions. All animals were fed under conditions of 25 °C and 55% of humidity and allowed free access to standard laboratory water and chow. To establish the xenograft 4T1‐tumor‐bearing mouse models, 4T1 breast cancer cells (5 × 10^5^) suspended in 100 µL PBS buffer were injected subcutaneously into the flanks of each mouse. After about 10 days, mice with tumor volumes at about 100 mm^3^ were used subsequently.

### In Vivo NIR Fluorescence Imaging

The in vivo fluorescence images were acquired on the commercial IVIS Spectrum imaging system (PerkinElmer). The xenograft 4T1‐tumor‐bearing mice were administered with TTFMN‐NPs in saline (10 mg TTFMN kg^−1^ mouse) through tail vein. Then, the fluorescence images of mice were obtained as a function of postinjection times (0, 1, 3, 6, 12, and 24 h). In an attempt to evaluate the tissue distributions of TTFMN‐NPs, the mice were sacrificed at 24 h postinjection. Major organs (heart, liver, spleen, lung, and kidney) and tumor were excised, followed by washing the surface with saline several times for NIR fluorescent imaging and quantitative analyses using the IVIS Spectrum imaging system.

### In Vivo Phototherapeutic Study

When the inoculated tumor grew to a size about 100 mm^3^, the xenograft 4T1‐tumor‐bearing mice were randomly classified as four groups (*n* = 5) and, respectively, received treatments of Saline, Saline + L, TTFMN‐NPs, and TTFMN‐NPs + L. For “Saline” and “TTFMN‐NPs” groups, 200 µL of saline and TTFMN‐NPs (10 mg TTFMN kg^−1^) were separately intravenously injected into the 4T1‐tumor‐bearing mice without subsequent white light irradiation. In case of “Saline + L” and “TTFMN‐NPs + L” groups, the white light irradiation (100 mW cm^−2^) at the tumor site in each group for 20 min was carried out after intravenous injection of saline and TTFMN‐NPs (10 mg TTFMN kg^−1^) for 12 h, respectively. Typical treatments were performed every three days, totally for 15 days, and the mouse body weight as well as tumor volume were also recorded every 3 days during 15 day study duration for courting the change of body weight and relative tumor volume. The tumor volume was measured by a vernier caliper and calculated as *V*  =  *a*  ×  *b* ^2^/2. (*a*: tumor length; *b*: tumor width). Relative tumor volume (RTV) was calculated as RTV  =  V/V_0_ (*V*_0_ was the initial tumor volume).

### Histological and Immunohistochemical Analysis

After complete treatment of 15 days, all the mice were sacrificed, and the tumors were dissected from the mice and weighed. Then, the tumor tissues were fixed in 4% v/v formalin overnight, embedded in paraffin, and sectioned at 5 µm thickness. After that, the slices were stained with H&E for histopathological evaluation. The apoptosis level of tumor cells was examined by using TUNEL staining. Monoclonal antibody Ki67 response was performed to detect proliferation activity of tumor cells. CD31 test was carried out to inspect new microvessels in tumor tissues. The slices were imaged by an inverted optical microscope.

### In Vivo Biosafety Evaluation

In order to estimate the potential systemic toxicity of TTFMN‐NPs, healthy BALB/c mice (4–5 weeks old) were administered with TTFMN‐NPs, at a dose of 10 mg TTFMN kg^−1^ mouse by intravenous injection. After 1 week, the blood samples of the mice were collected into blood collection tubes immediately by enucleation of mouse eyes before being sacrificed. The main hematological markers and serum biochemical parameters were further tested. Meanwhile, the major organs (heart, liver, spleen, lung, and kidney) were also dissected from the mice for further H&E analysis. As noted, the blood sample and major organs from the untreated mice were used as a control group to compare the biosecurity.

### Statistical Analysis

Experiments in this study were carried out at least 3 times. Data were expressed as the mean ± standard deviation (SD). One‐way analysis of variance (ANOVA) testing was used to determine the significance between experimental and control groups by GraphPad Prism 7, and a value of *p* < 0.05 was considered statistically significant.

## Conflict of Interest

The authors declare no conflict of interest.

## Supporting information

Supporting Information

## Data Availability

Data available on request from the authors.
