# Supplementary material for: Good Steel Used in the Blade: Well‐Tailored Type‐I Photosensitizers with Aggregation‐Induced Emission Characteristics for Precise Nuclear Targeting Photodynamic Therapy
Source: Adv Sci (Weinh). 2021 May 21;8(14):2100524. doi: 10.1002/advs.202100524 (PMC8292883; doi:10.1002/advs.202100524)
Supplement: Supplementary file 1 — Supporting Information [file ADVS-8-2100524-s001.pdf]

© 2021 Wiley-VCH GmbH

ADVANCED SCIENCE
Open Access

Supporting Information

for *Adv. Sci.*, DOI: 10.1002/advs.202100524

Good Steel Used in the Blade: Well-Tailored Type-I Photosensitizers with Aggregation-Induced Emission Characteristics for Precise Nuclear Targeting Photodynamic Therapy

Miaomiao Kang, Zhijun Zhang, Wenhan Xu, Haifei Wen, Wei Zhu, Qian Wu, Hongzhuo Wu, Junyi Gong, Zhijia Wang, Dong Wang, and Ben Zhong Tang**

Supporting Information

Good Steel Used in the Blade: Well-Tailored Type-I Photosensitizers with Aggregation-Induced Emission Characteristics for Precise Nuclear Targeting Photodynamic Therapy

Miaomiao Kang, Zhijun Zhang, Wenhan Xu, Haifei Wen, Wei Zhu, Qian Wu, Hongzhuo Wu, Junyi Gong, Zhijia Wang, Dong Wang, and Ben Zhong Tang**

Dr. M. Kang, Dr. Z. Zhang, H. Wen, Dr. W. Zhu, Dr. Q. Wu, Dr. H. Wu, Prof. D. Wang
Center for AIE Research, Shenzhen Key Laboratory of Polymer Science and Technology, Guangdong Research Center for Interfacial Engineering of Functional Materials, College of Materials Science and Engineering, Shenzhen University, Shenzhen, 518060, China
E-mail: wangd@szu.edu.cn
W. Xu, J. Gong, Prof. B. Z. Tang
Hong Kong Branch of Chinese National Engineering Research Center for Tissue Restoration and Reconstruction, Department of Chemistry, The Hong Kong University of Science and Technology, Clear Water Bay, Kowloon, Hong Kong, 999077, China
E-mail: tangbenz@ust.hk
Dr. Z. Wang
State Key Laboratory of Fine Chemicals, School of Chemical Engineering, Dalian University of Technology, Dalian 116024, China

Table of contents

Figure S1. The synthetic routes of TFMN and TTFMN. 3
Figure S2. ^{1}H NMR spectra of TFMN. 3
Figure S3. ^{13}C NMR spectra of TFMN. 4
Figure S4. HRMS spectrum of TFMN. 5
Figure S5. ^{1}H NMR spectra of TTFMN. 5
Figure S6. ^{13}C NMR spectra of TTFMN. 5
Figure S7. HRMS spectrum of TTFMN. 6
Figure S8. Frontier molecular orbitals for TFMN and TTFMN. 6
Figure S9. AIE curve of TFMN and corresponding digital images. 7
Table S1. Properties of AIEgens. 7
Figure S10. PL spectra of DCFH after different treatments. 8
Figure S11. Comparison in overall ROS production of TTFMN, Ce6 and RB. 8
Figure S12. $^•$OH generation of CV indicated by HPF. 9
Figure S13. Cyclic voltammograms of TFMN and TTFMN. 9
Figure S14. Gibbs free energy changes. 10
Figure S15. pH-dependent penetration of TTFMN-NPs. 10
Figure S16. The overlap coefficient between TTFMN-NPs and LysoTracker Blue inside cells. 10
Figure S17. Photostability test. 11
Figure S18. Quantitative fluorescence intensity of DCFH inside cells. 11
Figure S19. Biocompatibility evaluation to normal cells. 11
Figure S20. Live/dead cell staining of 4T1 cells after various treatments. 12
Figure S21. Immunofluorescent staining of 4T1 cells for DNA damage analysis. 12
Figure S22. Quantitative fluorescent curve of tumor tissues at different monitoring times. 13
Figure S23. *Ex vivo* NIR fluorescent images and corresponding quantitative analysis. 13
Figure S24. Body weight changes of tumor-bearing mice after 15-days treatments. 13
Figure S25. Blood biochemistry indexes of mice after different treatments. 14
Figure S26. H&E-stained slice images of major organs after different treatments. 14
Table S2. Routine blood indexes of mice after different treatments. 15

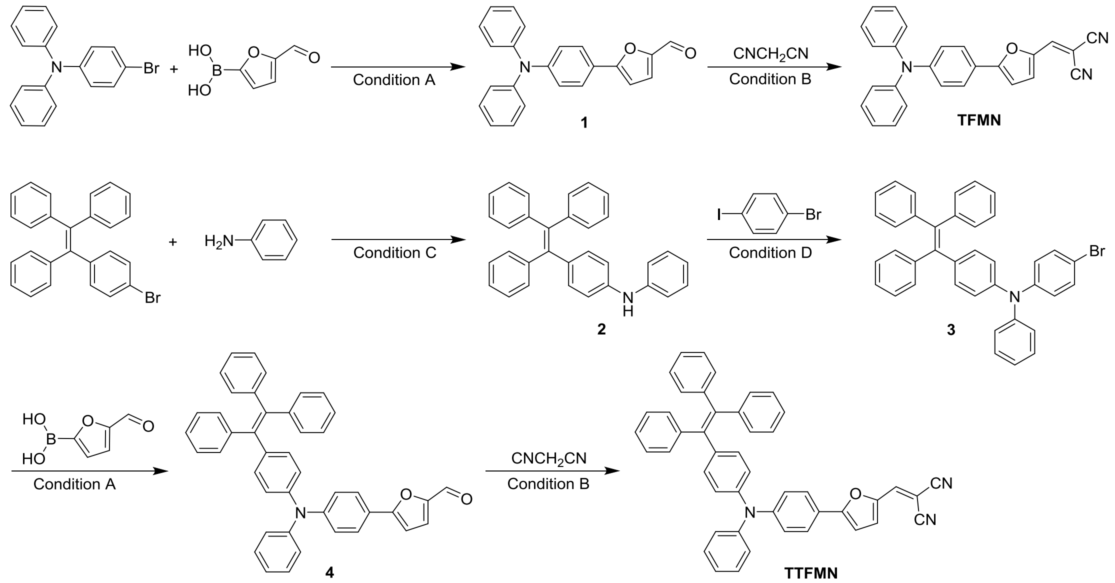

Figure S1. The synthesis routes of TFMN and TTFMN. Condition A: Pd(dppf)Cl_2, K_2CO_3, MeOH/Toluene, 75 °C, 16 h; Condition B: EtOH, reflux, 72 h; Condition C: $Pd_2(dba)_3$, P(*t*-Bu)$_3$, NaO*t*-Bu, Toluene, 120 °C, 24 h; Condition D: CuI, 1,10-phenanthroline, KOH, Toluene, 120 °C, 48 h.

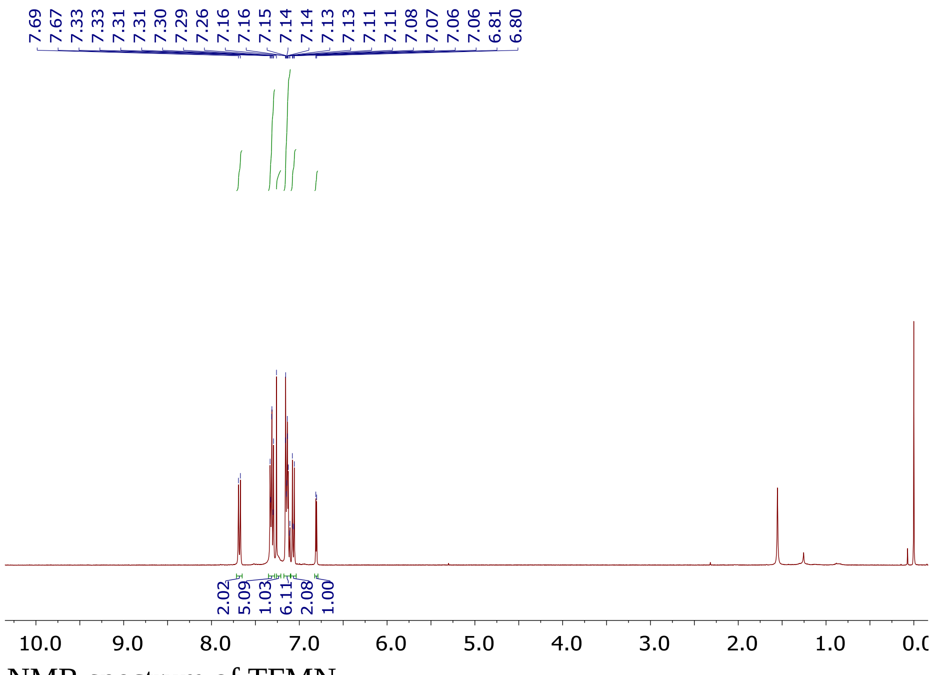

Figure S2. ^{1}H NMR spectrum of TFMN.

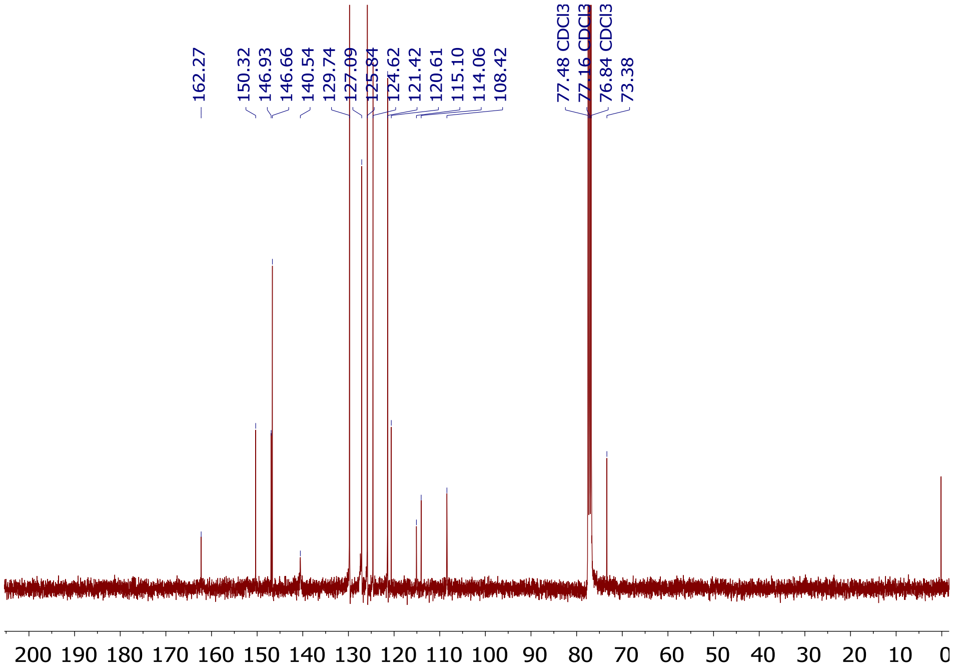

Figure S3. ^{13}C NMR spectrum of TFMN.

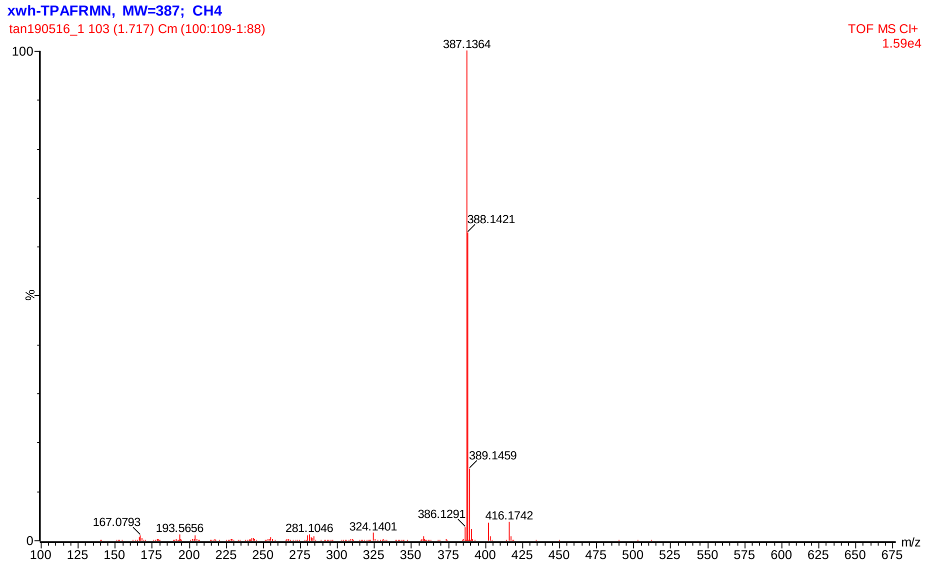

Figure S4. HRMS spectrum of TFMN.

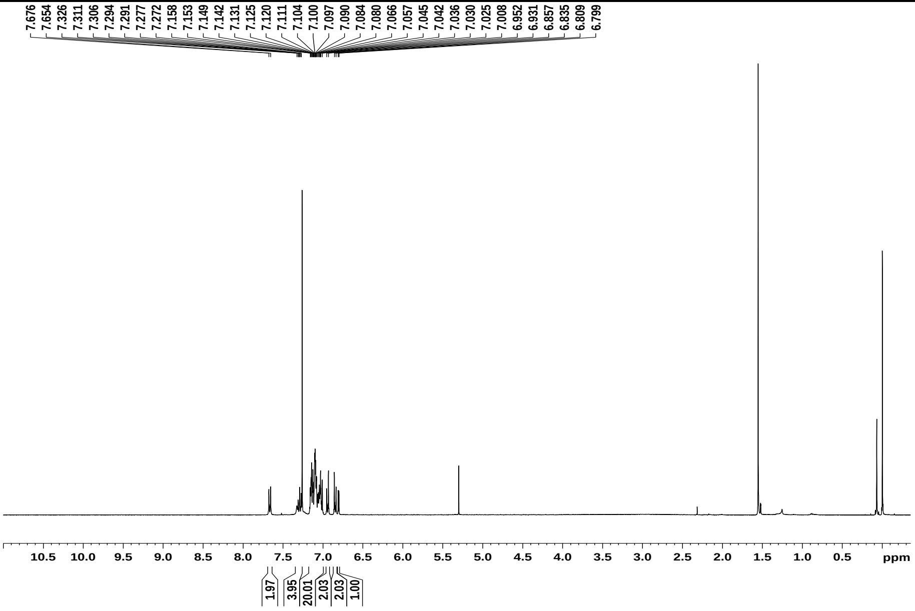

Figure S5. ^{1}H NMR spectrum of TTFMN.

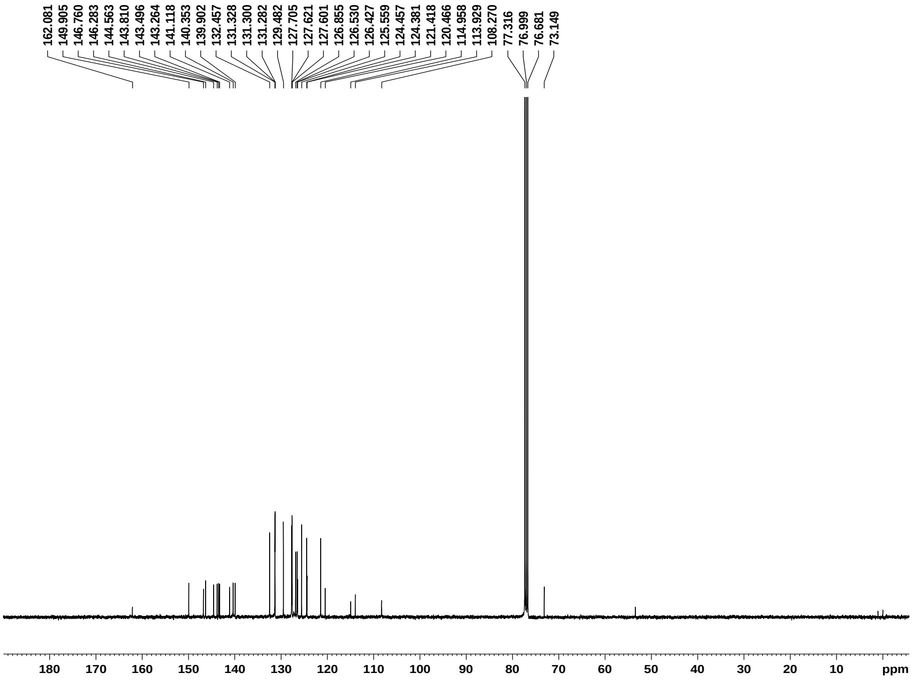

Figure S6. ^{13}C NMR spectrum of TTFMN.

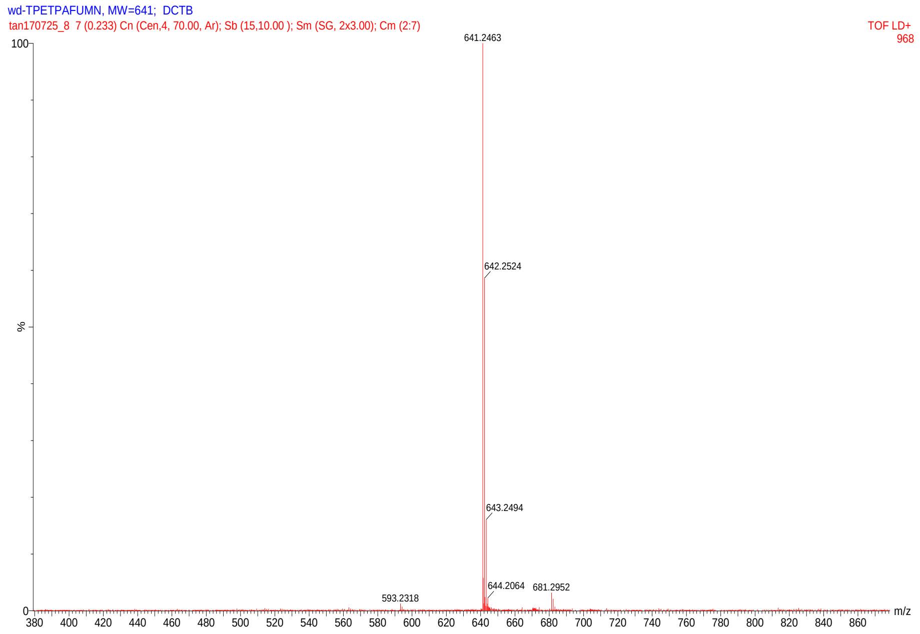

Figure S7. HRMS spectrum of TTFMN.

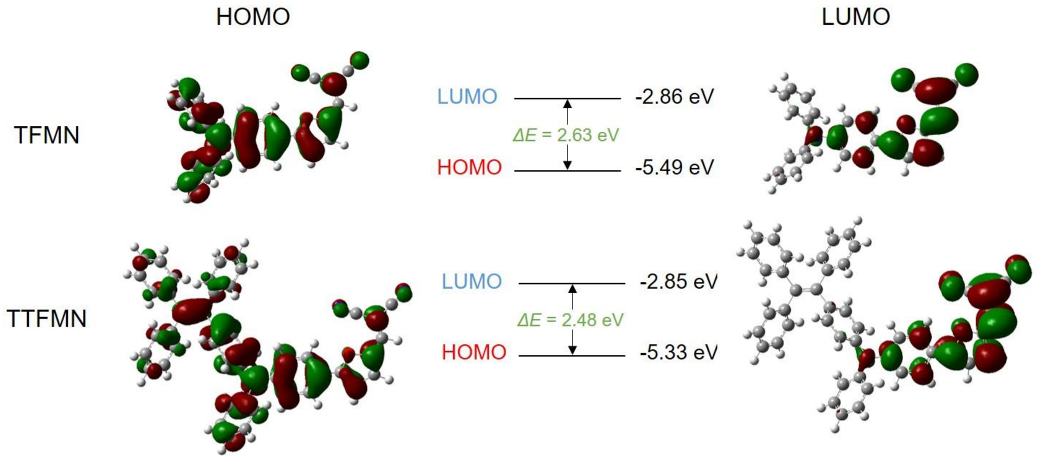

Figure S8. Frontier molecular orbitals for TFMN and TTFMN. Calculations were performed by density functional theory calculations at the B3LYP/6-311G** level using the Gaussian 09 program.

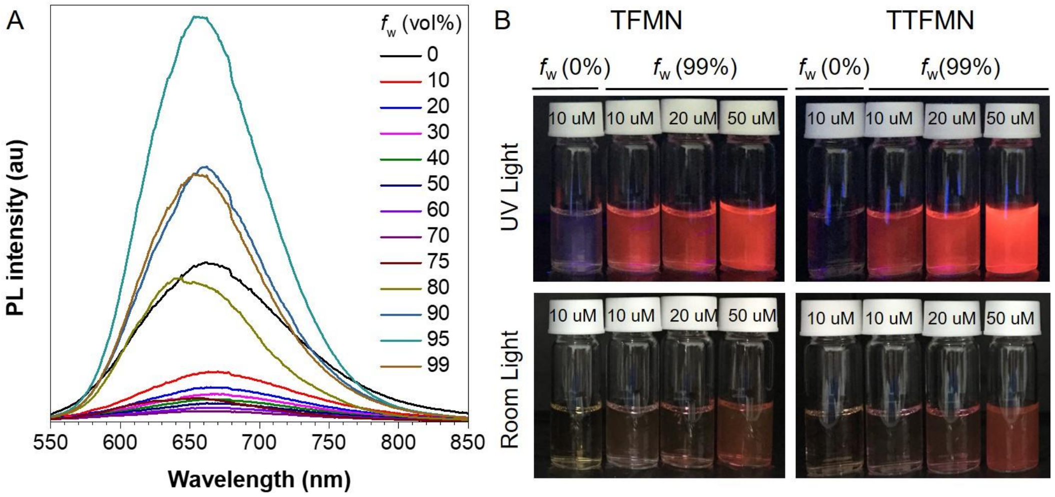

Figure S9. (A) PL spectra of TFMN (10 μM) in ACN/water mixtures with different water fractions (f_w). (B) Digital images of TFMN and TTFMN in pure ACN and ACN/water mixtures (f_w = 99%) at various concentrations.

Table S1. Properties of AIEgens.

AIEgens	$\lambda_{abs, soln.}$ [nm][a]	$\lambda_{em, soln.}$ [nm][b]	$\lambda_{em, aggr.}$ [nm][c]	$\lambda_{em, solid}$ [nm][d]	α_{AIE}[e]	$\Phi_{F, soln.}$ [%][f]	$\Phi_{F, aggr.}$ [%][g]	$\Phi_{F, solid}$ [%][h]	T[ns][i]	$\Delta E_{H\text{-}L}$ [eV][j]	$\Delta E_{s\text{-}t}$ [eV][k]
TFMN	482	662	655	651	2.6	1.6	2.7	3.2	1.64	2.63	0.24
TTFMN	490	664	661	664	105.2	0.4	4.3	16.9	2.52	2.48	0.20

[a]Absorption maximum in ACN solution; [b]Emission maximum in ACN solution; [c]Emission maximum in aggregate state; [d]Emission maximum in solid state; [e]$\alpha_{AIE} = I_{aggr,max} / I_{soln.}$; [f]Fluorescence quantum yield of AIEgens in ACN solution; [g]Fluorescence quantum yield of AIEgens in aggregate state; [h]Fluorescence quantum yield of AIEgens in solid state determined by a calibrated integrating sphere; [i]Lifetime of AIEgens; [j]The value of HOMO-LUMO energy gap of AIEgens; [k]The value of singlet-triplet energy gap of AIEgens.

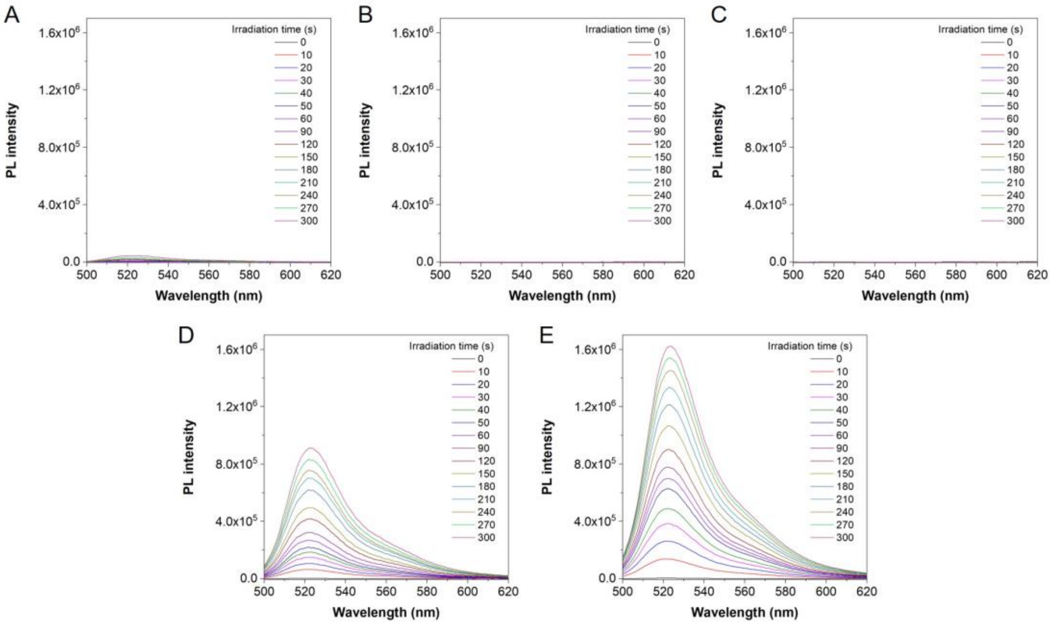

Figure S10. PL spectra of DCFH in PBS in present of (A) DCFH alone, (B) TFMN alone, (C) TTFMN alone, (D) TFMN+DCFH and (E) TTFMN+DCFH after exposure to white light irradiation with different time. The concentration of TFMN or TTFMN is 2 μM; Light power: 22.1 mW cm^{-2}.

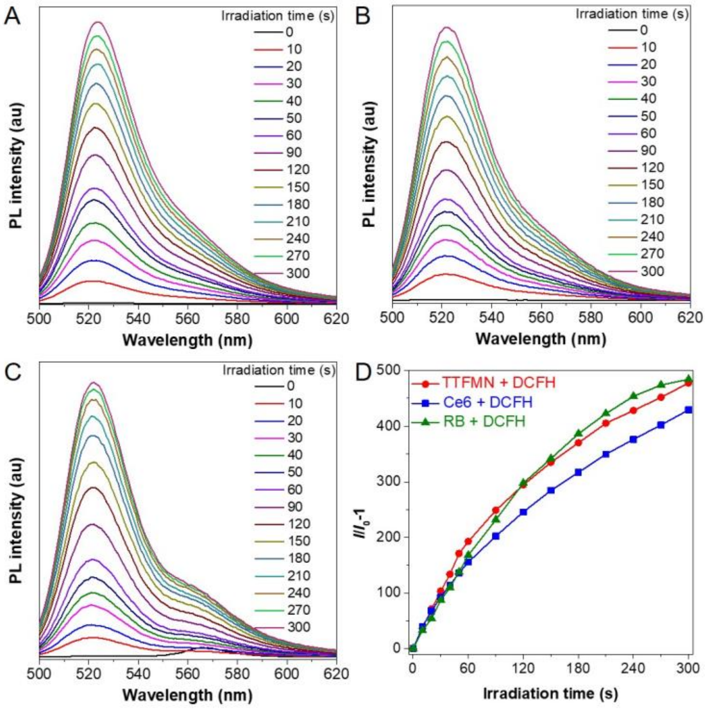

Figure S11. PL spectra of DCFH in PBS in present of (A) TTFMN, (B) Ce6 and (C) RB after exposure to white light irradiation with different time. (D) Relative changes in PL intensity of DCFH in A, B and C. I_0 and I are the PL intensity of DCFH before and after irradiation, respectively. The concentration of TTFMN, Ce6 and RB is 2 μM; Light power: 22.1 mW cm^{-2}.

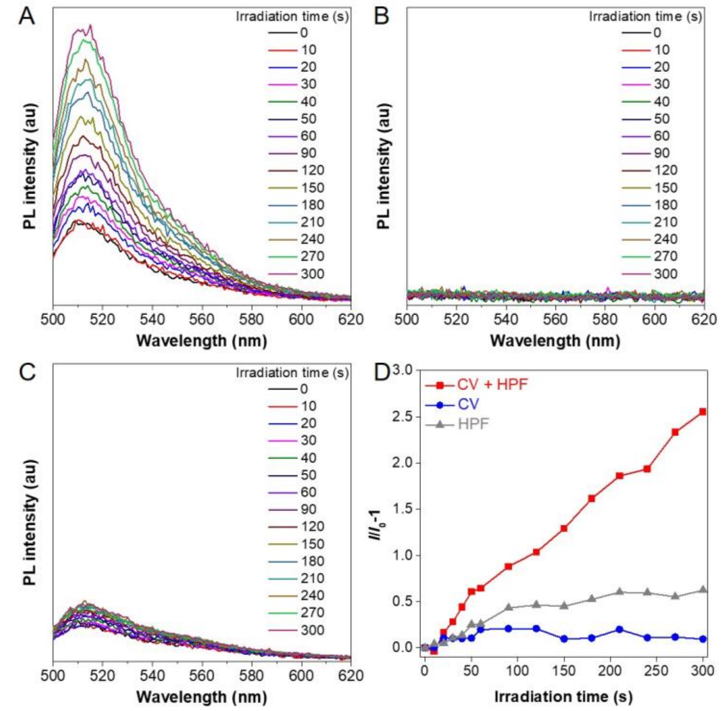

Figure S12. (A) PL spectra of HPF in PBS in present of CV, (B) PL spectra of CV in PBS, (C) PL spectra of HPF in PBS after exposure to white light irradiation (22.1 mW cm^{-2}) with different time. (D) Relative changes in PL intensity of A, B and C. I_0 and I are the PL intensity of HPF before and after irradiation, respectively. The concentration of CV is 2 μM.

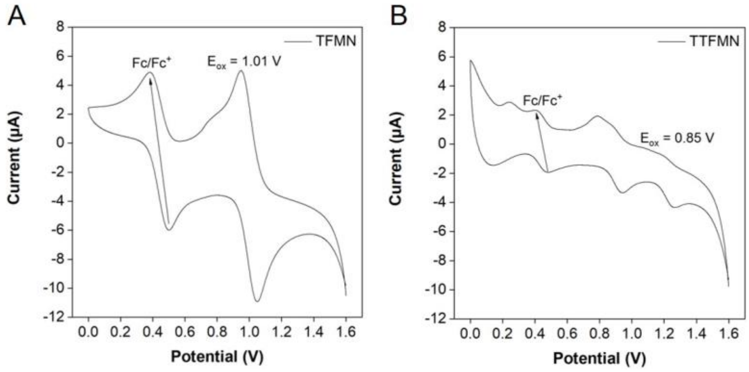

Figure S13. Cyclic voltammograms (CVs) of TFMN and TTFMN (1 mM) in CH_2Cl_2 solution. The CVs were determined by using 0.1 M *n*-Bu_4NPF_6 as the supporting electrolyte, saturated calomel electrode (SCE) as the reference electrode, Pt disk and Pt wire as counter electrodes, and ferrocene was used as internal standard. The scan rate was 50 mV s^{-1}.

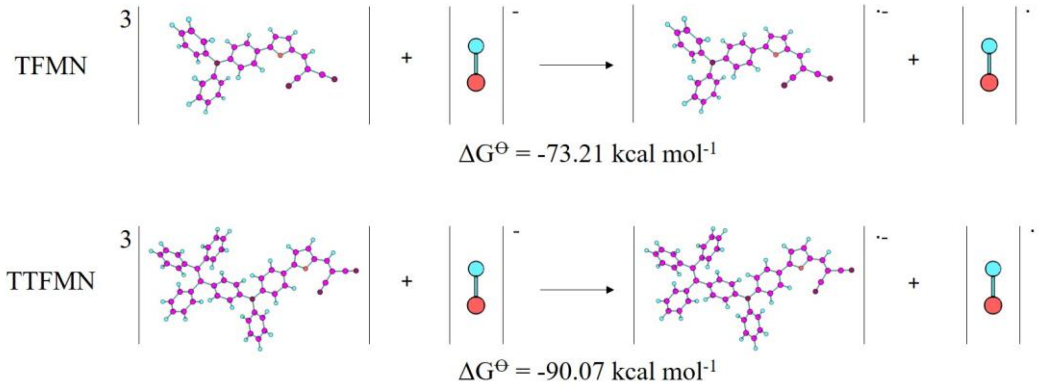

Figure S14. The Gibbs free energy changes of electron transfer between TFMN or TTFMN and hydroxyl anion calculated by PWPB95-D3/def2-TZVP using the quantum mechanism package ORCA 4.1.1.

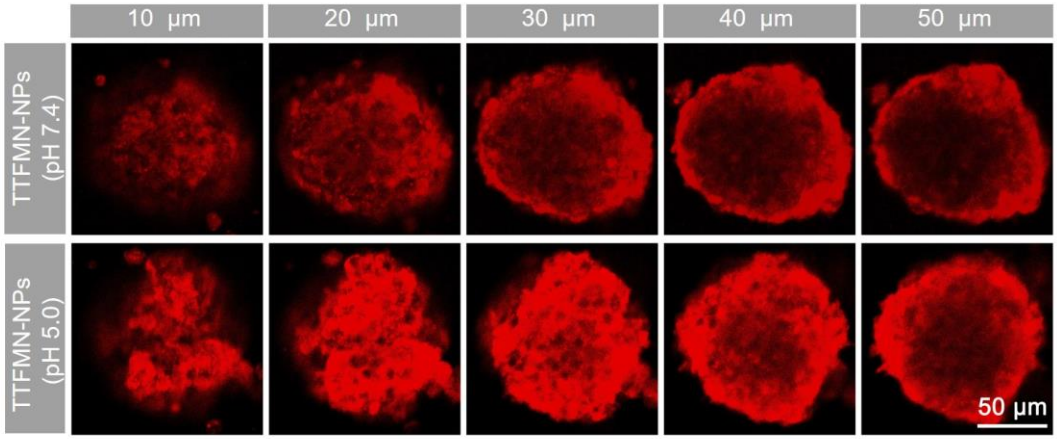

Figure S15. Representative CLSM images of pH-dependent penetration of TTFMN-NPs into 4T1 tumor spheroids model to different depths. The 4T1 tumor spheroids model were treated with TTFMN-NPs (10 μg mL^{-1}, preincubated in pH 7.4 or pH 5.0 PBS for 24 h) for 3 h.

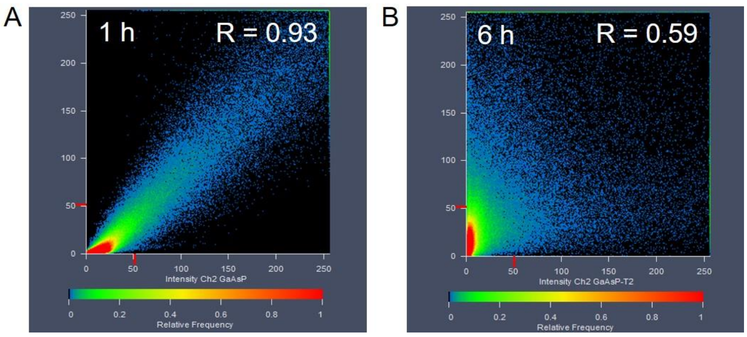

Figure S16. Scatter plots indicating the overlap coefficient of TTFMN-NPs and LysoTracker Blue inside 4T1 cells after being incubated with TTFMN-NPs (2 μg mL^{-1}) for (A) 1 h and (B) 6 h.

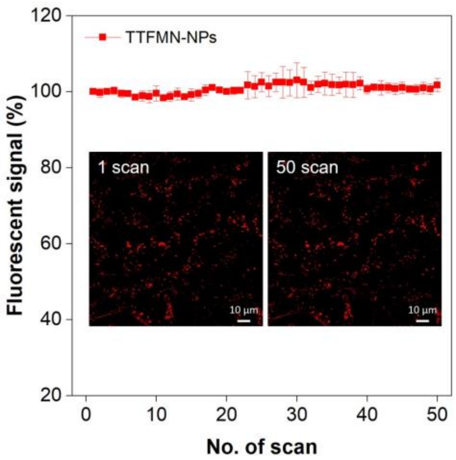

Figure S17. The plot of the fluorescent signal loss of 4T1 cells co-stained with TTFMN-NPs against the continuous laser irradiation (488 nm) scans by CLSM. Insert: CLSM images of 4T1 cells stained with TTFMN-NPs before and after 50 laser irradiation scans.

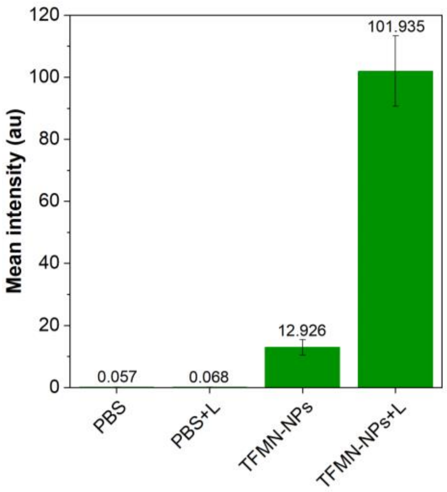

Figure S18. The corresponding mean fluorescence intensity of DCFH inside cells in different groups. Conditions: laser irradiation (488 nm, 2% power, 3 min), TTFMN-NPs (50 μg mL^{-1} TTFMN, 24 h).

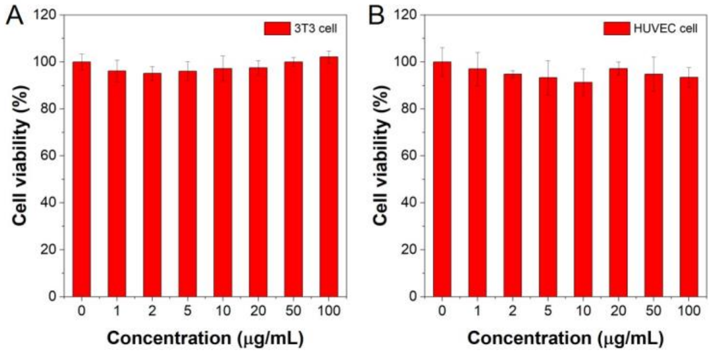

Figure S19. Cell viabilities of 3T3 and HUVEC cells after incubation with different concentrations of TTFMN-NPs for 48 h determined by MTT assay (mean ± SD, n = 6).

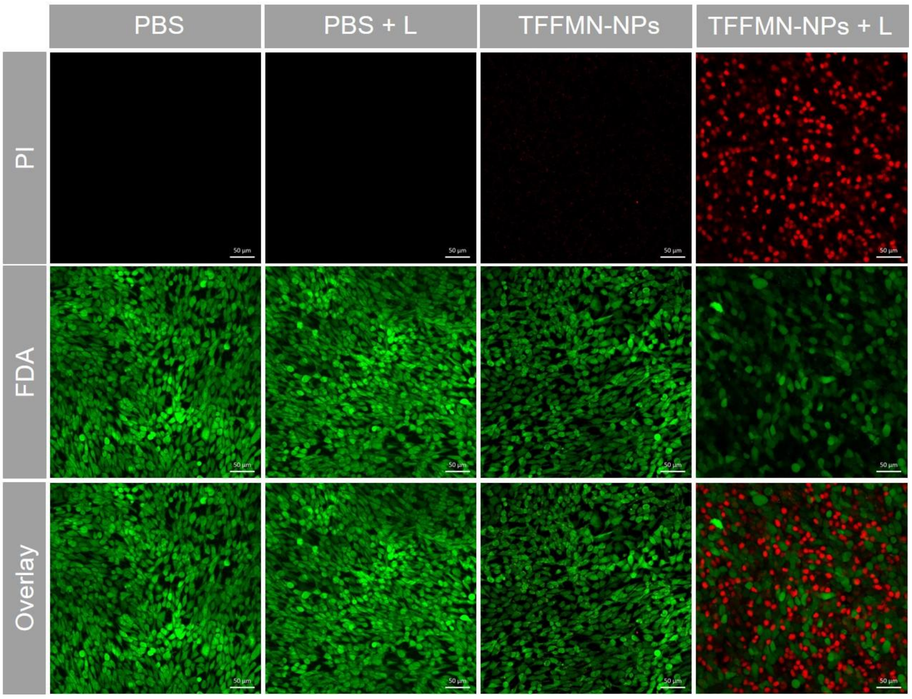

Figure S20. Live/dead cell staining of 4T1 cells after being treated with PBS, PBS + L, TTFMN-NPs, and TTFMN-NPs + L. The concentration of TTFMN-NPs is 50 μg mL^{-1} determined by TTFMN-NPs.

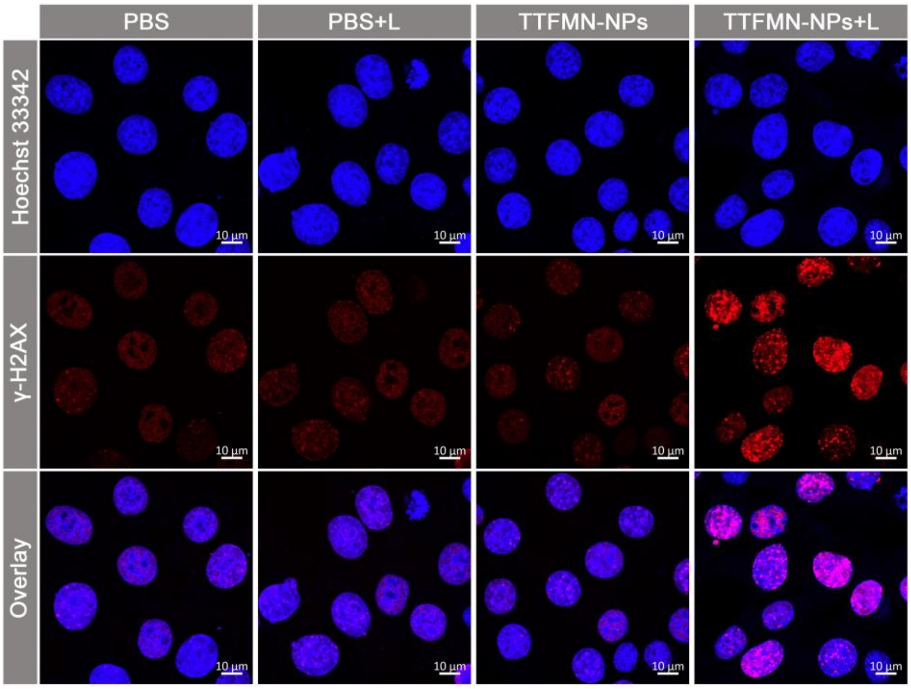

Figure S21. CLSM images of 4T1 cells stained with Hoechst 33342 and antibody against γ-H2AX after various treatments. White light irradiation (50 mW cm^{-2}) was conducted after cells were incubated with TTFMN-NPs (50 μg mL^{-1} TTFMN) for 24 h.

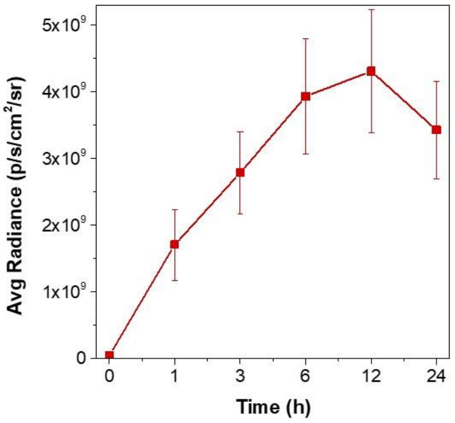

Figure S22. Quantitative fluorescent curve of tumor tissues at different monitoring times after intravenous injection with TTFMN-NPs (10 mg TTFMN kg^{-1}).

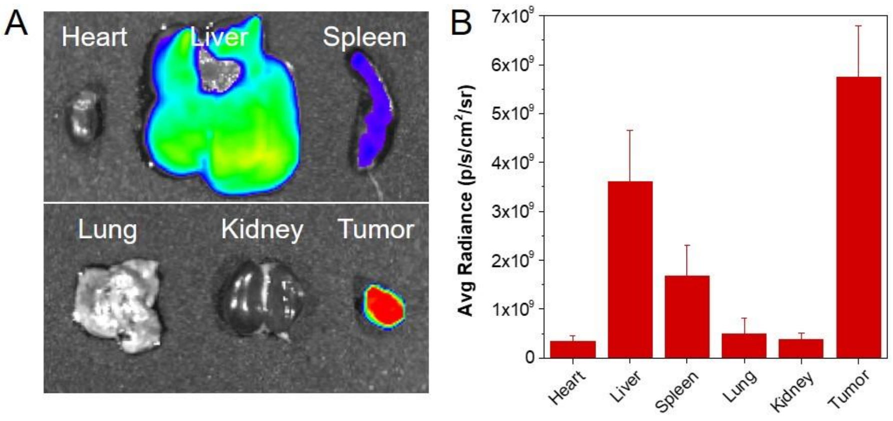

Figure S23. (A) *Ex vivo* NIR fluorescent images and (B) quantitative fluorescent intensity of tumor and major organs after intravenous injection with TTFMN-NPs (10 mg TTFMN kg^{-1}) for 24 h.

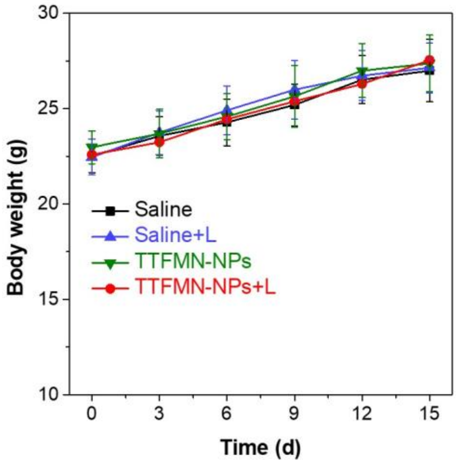

Figure S24. Body weight changes of 4T1 tumor-bearing mice with different treatments over 15 days.

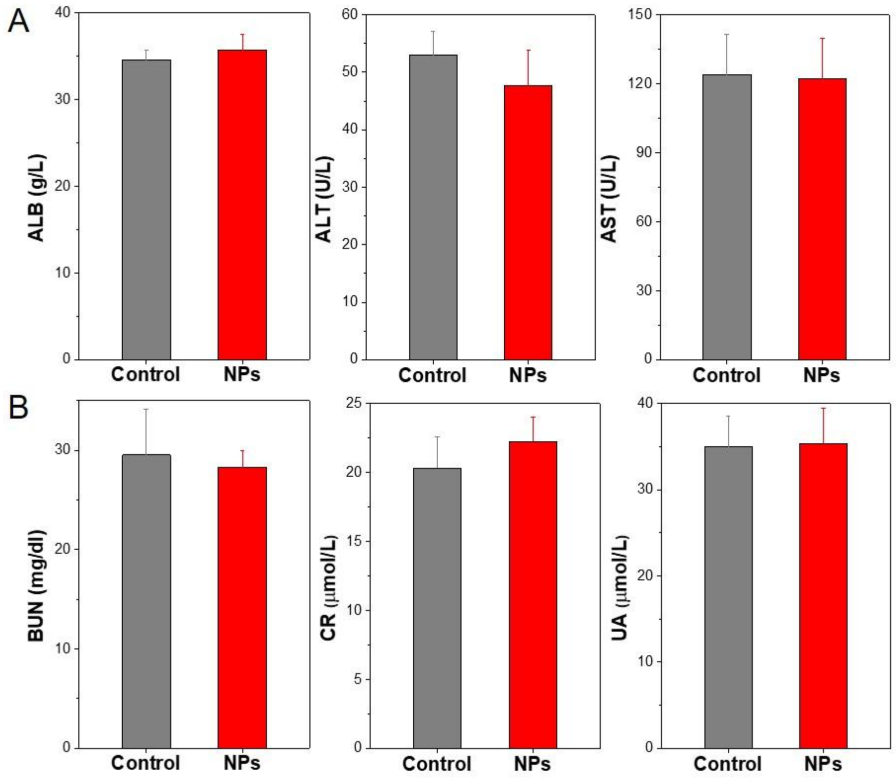

Figure S25. Blood biochemistry indexes including (A) hepatic function markers and (B) renal function markers of mice after intravenous injection with saline or TTFMN-NPs (10 mg TTFMN kg^{-1}) for 1 week.

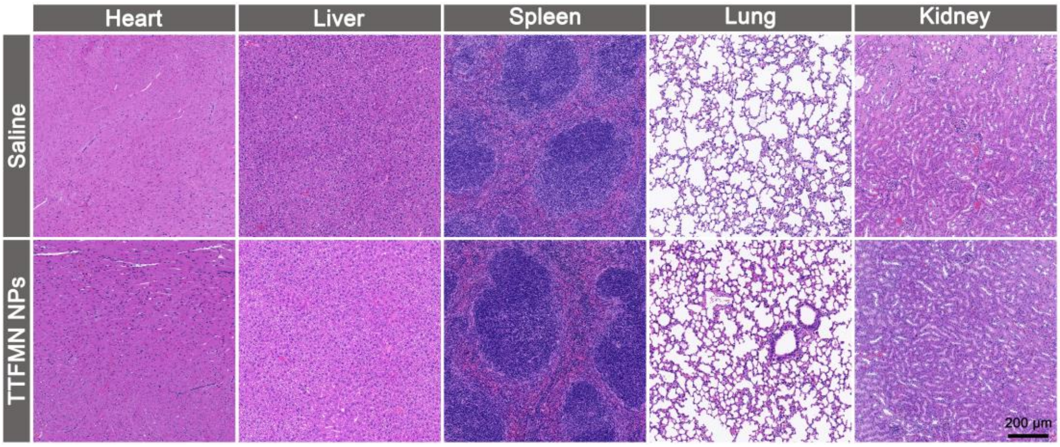

Figure S26. H&E-stained slice images of major organs of mice after intravenous injection with saline or TTFMN4-NPs (10 mg TTFMN kg^{-1}) for 1 week.

Table S2. Routine blood indexes of mice after different treatments.

		Control	TTFMN-NPs	Reference Range
WBC	(10^9/L)	3.8 ± 0.26	3.6 ± 0.76	0.8 ~ 6.8
Lymph	(10^9/L)	3.0 ± 0.79	2.7 ± 0.51	0.7 ~ 5.7
Mon	(%)	0.18 ± 0.05	0.16 ± 0.05	0.0 ~ 0.3
Gran	(10^9/L)	0.84 ± 0.17	0.77 ± 0.25	0.1 ~ 1.8
RBC	(10^{12}/L)	9.4 ± 0.23	9.2 ± 0.12	6.36 ~ 9.42
HGB	(g/l)	140.1 ± 4.7	141.2 ± 8.3	110 ~ 143
HCT	(%)	43.1 ± 0.87	41.4 ± 1.4	34.6 ~ 44.6
MCV	(fl)	46.9± 0.94	47.4 ± 0.61	48.2 ~ 58.3
MCH	(pg)	15.9 ± 0.35	16.0 ± 0.49	15.8 ~ 19
MCHC	(g/l)	334.6 ± 5.8	320.3 ± 9.2	302 ~ 353
RDW	(%)	16.0 ± 0.98	16.9 ± 0.52	13 ~ 17
PLT	(10^9/L)	1180.6 ± 342.5	1155.4 ± 329.2	450-1590
MPV	(fl)	5.5 ± 0.16	5.4 ± 0.11	3.8 ~ 6.0
